# *In Situ* X‐ray Diffraction Studies on the Reduction of U_3_O_8_ by Various Reducing Agents

**DOI:** 10.1002/chem.202500978

**Published:** 2025-05-27

**Authors:** Marvin Michak, Frank‐Constantin Ideker, Holger Kohlmann

**Affiliations:** ^1^ Faculty of Chemistry Institute of Inorganic Chemistry and Crystallography Leipzig University Johannisallee 29 04103 Leipzig Germany

**Keywords:** hydrogen, *in situ* investigations, reduction, uranium oxides

## Abstract

*In situ* X‐ray diffraction studies were conducted to elucidate the reduction pathways of U_3_O_8_ using various reducing agents, including hydrogen, the fluorinating agent polyvinylidene fluoride (PVDF), and calcium hydride (CaH_2_). The reduction processes were characterized by a transition from the orthorhombic, pseudo‐hexagonal phase α‐U_3_O_8_ to the hexagonal polymorph, followed by the formation of fluorite‐type UO_2+*x*_ phases. The reduction temperature and intermediate phases vary with the type of reducing agent, though they all follow a similar sequence of phases. In the presence of pure hydrogen and upon reaction with PVDF and CaH_2_, U_3_O_8_ transformed entirely into fluorite‐type phases UO_2+x_. By decreasing the chemical potential of hydrogen via its partial pressure, the behavior of U_3_O_8_ preceding the reduction to UO_2+*x*_ can be switched from an orthorhombic‐to‐hexagonal phase transition followed by disproportionation to two different U_3_O_8_ phases, presumably with different oxygen content, to a continuous oxygen loss within one U_3_O_8_ phase without prior phase transition. The detailed analysis of lattice parameters and phase transformations in the course of the investigated reactions offers insights into the reduction pathways of uranium oxides, highlighting the impact of different reducing environments on the reaction pathways and final products.

## Graphical abstract

The reduction of U₃O₈ by hydrogen, PVDF, and CaH₂ is investigated using continuous *in situ* X‐ray diffraction. Phase transitions, oxygen loss, and intermediate structures are analyzed. The study elucidates how different reducing environments influence reaction pathways and final products, offering new insight into uranium oxide chemistry relevant to nuclear material processing.

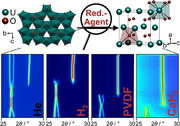

## 1 Introduction

Redox processes play an important role in the context of uranium oxide chemistry due to the actinide's capability to exist in various oxidation states. Uranium forms single valent oxides like the fluorite‐type UO_2_, containing tetravalent uranium, or several amorphous and crystalline phases of UO_3_, where uranium is purely hexavalent.^[^ ^1^ ^]^ Beyond that, also mixed‐valent oxides, like α‐U_3_O_8_ are known, where pentavalent and hexavalent uranium atoms are found in distinct crystallographic positions of the orthorhombic structure of this compound.^[^ ^1^, ^2^, ^3^, ^4^ ^]^

The world demand for U_3_O_8_ is estimated to 79,619 t in 2024, showing the substance's continuing importance for the nuclear industry worldwide, despite the growing skepticism toward the safety of nuclear energy sources.^[^ ^5^ ^]^ For many decades now, UO_3_ and U_3_O_8_ have therefore received much attention in research for their appearance in the nuclear fuel cycle, with U_3_O_8_ being the main component of the so‐called “yellowcake”–the first product after uranium extraction from its ores by sulfuric acid dissolution and following precipitation.^[^ ^6^, ^7^ ^]^ U_3_O_8_ is then refined to UO_3_, whose subsequent reduction by hydrogen gas plays an important role in this process, as the resulting UO_2_ is used as nuclear fuel or converted into UF_6_ for isotopic enrichment.^[^ ^6^, ^7^ ^]^

Therefore, the reduction reactions of UO_3_ and U_3_O_8_ to UO_2_ by hydrogen gas were already investigated several decades ago, focusing mainly on thermal analysis and X‐ray diffraction investigations on quenched samples, but also early *in situ* diffraction techniques were used on these systems. For the reduction of γ‐UO_3_ in the temperature range of 450–550 °C a stepwise reduction was proposed following the sequence^[^ ^8^ ^]^:

$$UO_{3}\overset{H_{2}}{\to }U_{3}O_{8+x}\overset{H_{2}}{\to }U_{3}O_{8}\overset{H_{2}}{\to }U_{3}O_{8-x}\overset{H_{2}}{\to }UO_{2}$$

In an early *in situ* investigation on the reduction of UO_3_ and U_3_O_8_, these results were confirmed, however without crystal structure refinement and thus without detailed structural information.^[^ ^9^ ^]^ It was added that at higher temperatures UO_2+*x*_ forms intermediately and is then gradually transformed to stoichiometric UO_2_, while for lower temperatures the phase formed is already close to UO_2_ as deduced from the reflection positions.^[^ ^9^ ^]^ These results agree with an earlier study on the reduction kinetics of U_4_O_9_.^[^ ^10^ ^]^ Later thermogravimetric reduction studies under different hydrogen concentrations also found the stepwise reduction for both oxides made from calcination of ammonium diuranate.^[^ ^11^ ^]^ Detailed kinetic studies on the reduction of U_3_O_8_ by hydrogen using thermogravimetry were interpreted by means of a nucleation–growth mechanism limited by the reactive desorption of water from the particle surface of U_3_O_8_ and UO_2_.^[^ ^12^ ^]^ In contrast to the findings for the reduction reactions, the oxidation of UO_2_ proceeds via more intermediate phases, including β‐U_4_O_9_ and β‐U_3_O_7_ as more recently investigated by *in situ* neutron powder diffraction of the isothermal oxidation.^[^ ^13^ ^]^ Photoelectron spectroscopy on thin films of UO_3_ showed the formation of surface hydroxyl groups by proton transfer to the surface's oxygen atoms.^[^ ^14^ ^]^

These results on the reduction reactions of uranium oxides paint the picture of a complex situation to which we herein contribute continuous *in situ* X‐ray diffraction measurements and sequential refinements of the crystal structures of oxides appearing in these processes. *In situ* methods offer great potential to create a more detailed understanding of crystal structures and reaction pathways of reactions under nonambient conditions.^[^ ^15^ ^]^ So far, to the best of our knowledge, none of the processes listed above was investigated by time resolved continuous *in situ* X‐ray powder diffraction (XRPD) applying a varying temperature program, which is why we use this technique in this contribution.

**Figure 1:**
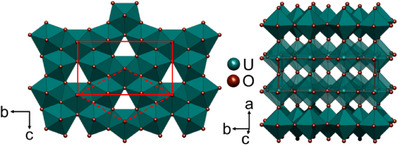
Crystal structure of α‐U_3_O_8_ (*Amm*2). The pseudo‐hexagonal structure consists of layers of edge‐sharing distorted pentagonal bipyramids of oxygen around the two crystallographically distinct uranium atoms. These layers are stacked along the *a*‐axis such that the pentagonal bipyramids are connected via a common corner. The solid red lines indicate the cell of the α‐modification, while the dashed lines show the cell of the hexagonal high‐temperature β‐modification ($P\bar{6}2m$) for the case that *b*/*c* = √3.

As a reference, the behavior of U_3_O_8_ in air and under an inert gas atmosphere is investigated similarly. It was earlier stated that the orthorhombic phase α‐U_3_O_8_ (space group *Amm*2, Figure 1) undergoes a gradual phase transition to a hexagonal polymorph crystallizing in a very similar structure of space group type $P\bar{6}2m$.^[^ ^16^ ^]^ The temperature at which the transition is completed was found to be 350 °C indicated by fully merged reflections in an early *in situ* X‐ray diffraction investigation on this process, with the hexagonal form persisting to 875 °C, before the phase starts to lose more oxygen and a splitting of the reflections is again observed.^[^ ^16^ ^]^ However, it is found that the theory of second‐order phase transitions cannot be applied in this case.^[^ ^17^ ^]^ The resulting product UO_2_ shows a strong nonstoichiometry toward a higher oxygen content (UO_2+*x*_). This results in the lattice parameter of the fluorite‐type structure shrinking significantly with a larger oxygen content, spanning a range from 5.4706 Å for *x* = 0 to 5.4453 Å for *x* = 0.224, as determined by X‐ray diffraction and density measurements.^[^ ^18^ ^]^

It is further known that the reaction of rare earth oxides *RE*_2_O_3_ (*RE* = rare earth element) with CaH_2_ and fluorinated polymers like polyvinylidene fluoride (PVDF) yields heteroanionic oxides, specifically hydride oxides *RE*HO and oxide fluorides *RE*OF.^[^ ^19^, ^20^, ^21^ ^]^ We were interested in whether uranium oxides would also form such compounds as a product or intermediate, which is why we also investigated their reaction with these reducing agents via *in situ* X‐ray diffraction. By comparing these observations to those made from classical reduction reactions by hydrogen and decomposition in air or under an inert gas atmosphere, we would like to provide insights into the differences between the respective reaction pathways and intermediate phases formed therein.

## 2 Materials and Methods

### 2.1 Sample Preparation

Hydrogen gas (H_2_, Air Liquide, 99.9%), hydrogen‐argon mixture (ARCAL 15, Ar + 5 vol.% H_2_, Air Liquide), helium (He, Air Liquide, 99.999%), polyvinylidene fluoride (PVDF; powder, Apollo Scientific), hydrogen peroxide (35%, OQEMA), nitric acid (chemically pure, 65%, OQEMA), and *n*‐pentane (Carl Roth, 95%) were used as received commercially.

CaH_2_ was prepared by reacting freshly distilled calcium metal (Alfa Aesar, distilled at 10^−5^ mbar and 1173 K) with 90 bar hydrogen gas (H_2_, Air Liquide, 99.9%) at 673 K for 36 hours in a home‐built autoclave made from alloy Böhler L718V (Inconel). U_3_O_8_ was prepared by the combustion of uranyl peroxide UO_4_∙2H_2_O in air.^[^ ^22^ ^]^ Uranyl peroxide was previously prepared by dissolving a piece of uranium metal in boiling concentrated nitric acid (chemically pure, 65%, OQEMA). The nitric acid was subsequently evaporated and the residual, yellow solid dissolved in boiling water. To the boiling solution, H_2_O_2_ solution (35%, OQEMA) was slowly added, quickly resulting in the precipitation of a pale yellow solid. After completion of the precipitation (indicated by an almost colorless solution and no further precipitation upon addition of H_2_O_2_), the solid was filtered off, washed three times with water, and dried by washing with diethyl ether. Finally, the substance was heated to 1023 K in air and held there for 12 hours using a chamber furnace before letting it cool to room temperature with its natural cooling rate after turning off the heating. The resulting olive green powder was shown to be single‐phase orthorhombic U_3_O_8_ by XRPD.

### 2.2 X‐ray Powder Diffraction

*In situ* X‐ray diffraction experiments were performed on a SmartLab diffractometer (Rigaku, Tokyo, Japan) equipped with a HyPix‐3000 2D semiconductor detector using ${\text{Cu-K}}_{\bar{\alpha }}$ radiation. For flat specimen measurements, the samples were placed in an XRK900 reaction chamber (Anton Paar, Graz, Austria) with attached gas control before temperature dependent measurements were performed in Bragg‐Brentano (BB) or Parallel Beam (PB) geometry with *θ*‐*θ*‐mode. On the primary site, a CBO optics and an incident slit of 10 mm were used, while on the secondary site, a nickel foil of 15 µm was attached as a K*_β_*‐filter, when using BB geometry. For measurements under gas flow, the chamber was evacuated for 45 minutes before flushing with the corresponding gas three times. During the measurements, a pressure of 1.0–1.3 bar was maintained by an overpressure valve. A gas flow of 10 sccm was kept by a 300 Vue mass flow controller (Teledyne Hastings Instruments, Hampton, US) in the reactions with hydrogen and argon/hydrogen mixture.

For the capillary sample measurement of U_3_O_8_ and CaH_2_, the sample was mixed with about 85–90 wt.% of diamond powder (synthetic diamonds, mesh size 500/600, Onyxmet) to reduce X‐ray absorption and placed in a graphitized quartz glass capillary of 0.5 mm inner diameter in an argon‐filled glovebox (maintaining oxygen and moisture levels of the atmosphere at < 1.0 ppm) before sealing the capillary shut with an oxyhydrogen torch. The samples were subsequently placed in an HTK1200 reaction chamber (Anton Paar, Graz, Austria) before temperature dependent measurements were conducted in PB geometry with 2*θ*‐mode. The measurements on capillary samples were conducted with a parallelized X‐ray beam and hence without a K*_β_*‐filter. The exact measurement conditions of every dataset are presented in Table 1. The *in situ* experiment investigating the behavior of pure U_3_O_8_ in a dynamic vacuum was performed in the same HTK1200 reaction chamber, applying a dynamic vacuum and using the flat specimen setup of the reaction chamber.

**Table 1:** Measurement conditions of the X‐ray diffraction data discussed herein.

U_3_O_8_ + …	Optics	Incident slit height	Incident slit width / mm	Scan speed / °/min	2*θ*–range
**Air**	BB	1/3 °	10	3.5	14°–80°
**He**	BB	1/3 °	10	3.5	14°–80°
**H_2_**	PB	1 mm	5	9	10°–90°
**Ar/H_2_**	PB	4 mm	10	5	10°–90°
**PVDF**	PB	0.3 mm	10	5	10°–80°
**Vacuum**	PB	0.6 mm	10	5	10°–90°
**CaH_2_**	PB	0.6 mm	10	1.5	20°–40°

Abbreviations: BB, Bragg‐Brentano geometry, divergency slit; PB, parallel beam.

Crystal structures were sequentially refined using the Rietveld method with a fundamental parameters approach and full profile analysis by the software TOPAS version 5 (Bruker AXS).^[^ ^23^, ^24^, ^25^ ^]^ The backgrounds were described by a Chebyshev polynomial of 10^th^ order. Zero‐point error, scaling factor, lattice constants, as well as crystallite size and strain were refined for all phases. Atomic parameters were refined for all uranium and calcium atoms and for oxygen atoms where possible. In some cases, especially for intermediate phases with low mass fractions, oxygen positions were kept at literature values to avoid nonsensical positions. For U_3_O_8_ in all measurements except the reduction by CaH_2_, anisotropic line broadening was described by the Stephens model for the orthorhombic crystal system to improve reflection profiles.^[^ ^26^ ^]^ Due to the samples’ usually high absorption coefficients, intensities were corrected by a surface roughness model in the case of flat samples and by a cylindrical sample model in the case of capillary sample environment.^[^ ^27^, ^28^ ^]^

## 3 Results and Discussion

**Figure 2:**
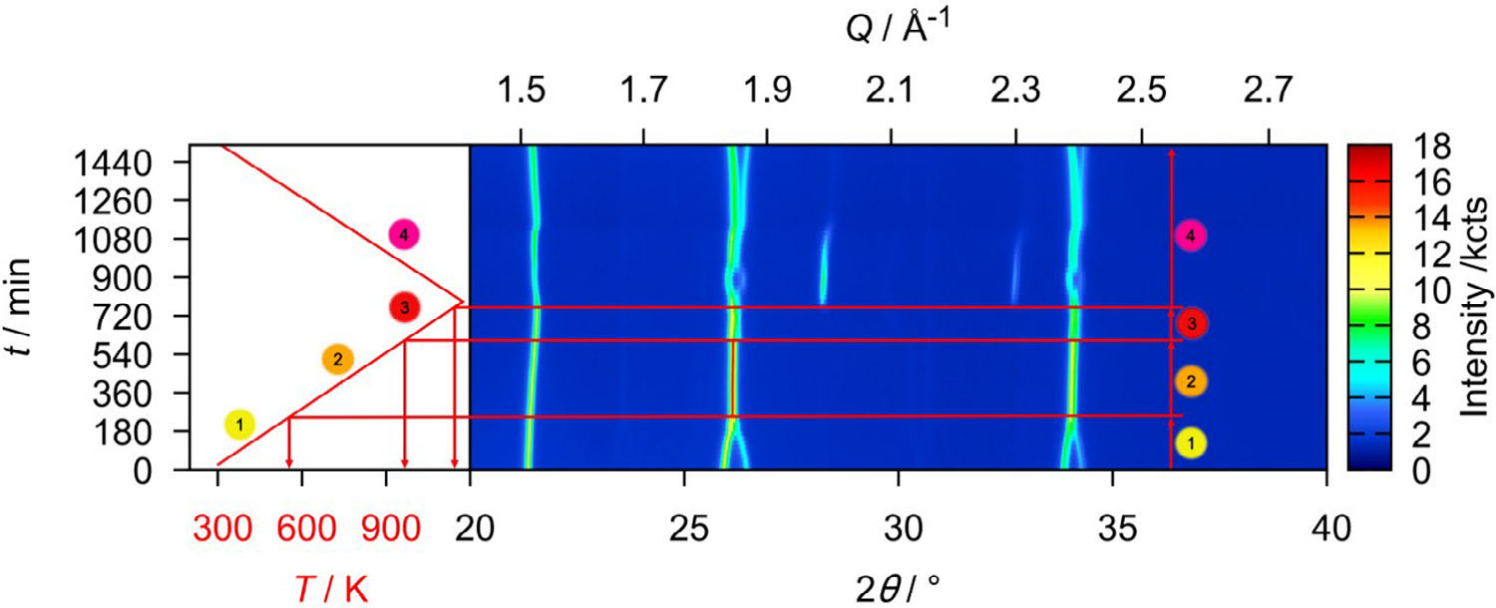
False‐color plot of part of the *in situ* XRPD (${\text{Cu-K}}_{\bar{\alpha }}$ radiation, BB geometry) data of U_3_O_8_ between room temperature and 1173 K in a static atmosphere of air. During the heating period, three phases of the reaction can be observed: 1. Until 572 K the orthorhombic phase gradually approaches hexagonal symmetry. 2. Upon further heating, the hexagonal phase thermally expands. 3. At 997 K, the formerly fused reflections suddenly split again, indicating a phase change likely caused by the beginning loss of oxygen. 4. The splitting of the reflections becomes more pronounced at 1147 K as a second phase appears which can be explained by a fluorite‐type phase UO_2+*x*_; this phase disappears upon further cooling.

As reference measurements, a sample of U_3_O_8_ was heated to 1173 K in a static atmosphere of air and helium, respectively, and monitored by *in situ* XRPD. A heating rate of 30 Kmin^−1^ was applied and XRPD data were recorded every 25 K to a temperature of 1073 K, before cooling at the same rate and recording XRPD data every 25 K until room temperature was reached. An exposure time of 15 minutes was applied for each measurement. The result of this experiment is depicted in Figure 2 as for all following *in situ* plots of diffraction data: The diffraction angle is found on the *x*‐axis and is shown against the time of the measurement. The temperature at each time can be concluded from the temperature profile found in the left‐hand part of Figure 2. For each diffractogram, the intensity is expressed by a false color, where blue depicts a low and red a high intensity.

In this case, the phase transition from the orthorhombic to the hexagonal modification mentioned above can very well be observed in the temperature range from room temperature to 572 K, where it is completed as indicated by the complete fusion of formerly split reflections, for example 013 and 002 at a diffraction angle of around 26°. Upon further heating, the hexagonal phase expands thermally before 997 K, the reflections suddenly split again, indicating the beginning of a loss of oxygen, stabilizing the orthorhombic modification.^[^ ^9^ ^]^ After more time at elevated temperatures, but already in the cooling process, the reflections split even more, as a second fluorite‐type phase UO_2+*x*_ appears.

This phase disappears again at further cooling, where it is reoxidized to orthorhombic U_3_O_8_, which also depicts the final product of this experiment.

**Figure 3:**
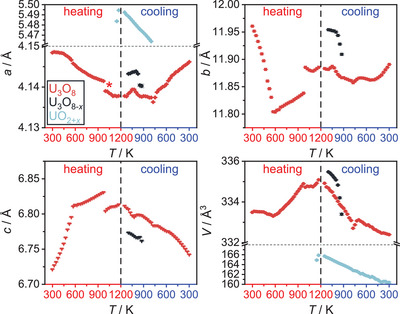
Evolution of the lattice parameters and the unit cell volume of all phases observed in the *in situ* X‐ray diffraction investigation of U_3_O_8_ in air for temperatures up to 1173 K. The red symbols denote the evolution of U_3_O_8_, the black symbols denote a second phase of U_3_O_8_ structure found to exist simultaneously in this temperature range (U_3_O_8‐*x*_), the blue symbols denote a fluorite‐type phase named UO_2+*x*_. At around 1000 K, a kink in lattice parameters hints toward the formation of a different phase, which was marked with an asterisk (*). Details on the treatment of this phase can be found in the text.

A sequential Rietveld refinement of the crystal structures of the respective phases throughout the experiment was conducted using only the orthorhombic phase U_3_O_8_ as the starting model for refinement. The results of the evolution of the lattice parameters of all phases observed in this refinement are depicted in Figure 3. The phase transformation from orthorhombic to hexagonal U_3_O_8_ is preceded by an anisotropic thermal change of lattice parameters, with *a* and *b* contracting and *c* expanding, which is in good agreement with literature, although we find a lower temperature of completion at 572 K as compared to the earlier proposed temperature of 623 K.^[^ ^16^ ^]^ The anisotropy in thermal expansion is rooted in the anisotropic connectivity of the [UO_7_] pentagonal bipyramids, that are edge‐sharing in layers spanning the *b*‐*c* plane, while they are only connected by common corners along *a* (Figure 1). Thereby, the ratio *b*/*c* approaches a ratio of √3, which makes it possible to index the resulting cell hexagonally. The phase transition can be easily identified by a kink in the lattice parameter versus temperature curves (Figure 1). At a temperature of 997 K a sudden jump in lattice constants appears, where the *a* and *c* lattice parameters become significantly smaller and *b* larger, the change in unit cell volume, however is rather small. This jump could be an indication of the beginning loss of oxygen, resulting in a stabilization of the orthorhombic‐pseudohexagonal structure of U_3_O_8_.^[^ ^9^ ^]^ Additionally, numerous new rather broad reflections of very low intensity appear, suggesting a sudden change in the ordering situation of the oxygen atoms, for example formation of a superstructure. Still, the diffraction pattern was best described by refining a jump in lattice parameters of orthorhombic U_3_O_8_, rather than by any known structure of possible ordering variants, superstructures, or different structure types of other binary uranium oxides containing less oxygen mentioned in literature (U_13_O_34_,^[^ ^29^ ^]^ U_3_O_7_,^[^ ^30^ ^]^ U_2_O_5_,^[^ ^31^ ^]^ or U_4_O_9_^[^ ^32^, ^33^, ^34^ ^]^). The phase was marked with an asterisk (*) in Figure 3. Although it might be a different phase, it will not be treated separately from U_3_O_8_ and was not addressed in further detail in the following investigations (it is also observed in the experiments investigating the behavior of U_3_O_8_ under helium and in a dynamic vacuum). Thermal displacement and positional parameter of the oxygen atoms had to be kept fixed in order not to become nonsensical, which also points toward a fundamental problem with the structure model. Since the change only appeared for a few measurements, the structure model of orthorhombic U_3_O_8_ was continued to be used at this point and the additional reflections were ignored, which resulted in more consistent refinements. It should therefore be noticed that the situation in this temperature range (997 ≤ *T* ≤ 1172 K) is likely to be much more complex, which cannot be sufficiently explained by evaluation of these data. Shortly after this transformation, a fluorite‐type structure is formed, which in earlier experiments of the reduction of U_3_O_8_ by hydrogen gas was identified to be an oxygen‐rich UO_2_ phase (UO_2+x_). This further supports the assumption of the U_3_O_8_ being thermally reduced by loss of oxygen.

**Figure 4:**
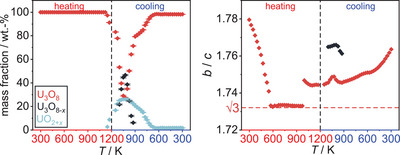
Evolution of the mass fractions of all phases observed in the *in situ* X‐ray diffraction experiment of U_3_O_8_ in air.

**Figure 5:**
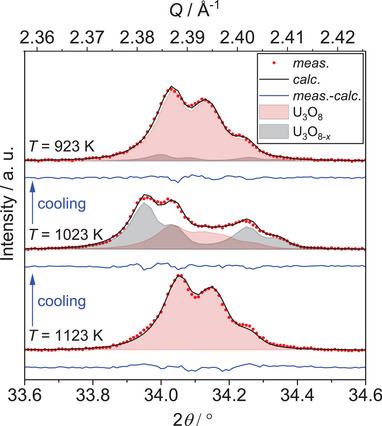
Selected Rietveld refinements based on the XRPD data around the 131 and 102 reflections of U_3_O_8_ during cooling in the temperature range of 1123 K to 923 K in air. The temperature decreases in steps of 100 K from bottom to top. The area filled black indicates the corresponding reflections of a second U_3_O_8_‐type phase, showing its intermediate formation. Bragg markers were omitted for clarity.

Upon further cooling of the material, the evolution of the individual lattice parameters of U_3_O_8_ becomes more complex, while the unit cell volume decreases smoothly. Regarding the mass fractions of the phases, the phase UO_2+_ *_x_* starts to vanish with further cooling from 1024 K, while simultaneously the mass fraction of U_3_O_8_ rises again, pointing toward a reoxidation of the UO_2+_ *_x_* due to the still elevated temperatures (Figure 4). Within this process, in the temperature range between 1099 K and 924 K, two simultaneously existing phases in U_3_O_8_ structure type were observed. The intermediately appearing phase shows significantly different lattice constants and an enlarged unit cell volume, pointing to a less pseudo‐hexagonal symmetry presumably caused by an oxygen deficiency as compared to U_3_O_8_, which is why in the following it will be called U_3_O_8‐x_ (Figure 5). The notation as U_3_O_8‐_ *_x_* could of course also be applied to the phase denoted as U_3_O_8_, since it is also a nonstoichiometric variant of the compound as indicated by the jump in lattice parameters due to a loss of oxygen as discussed above. However, for simplicity this phase is still being called U_3_O_8_ to differentiate it more easily from the isotypic phase that appears simultaneously in the respective temperature range and because it evolves continuously from the starting material. This phase could be an intermediate in the reoxidation process of UO_2+_ *_x_* to U_3_O_8_, since its mass fraction is highest before a decrease in phase fraction of UO_2+_ *_x_* and a gain in phase fraction for U_3_O_8_ is observed.

**Figure 6:**
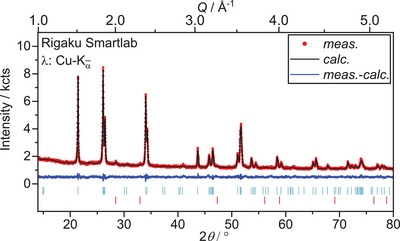
Rietveld refinement of the crystal structures of U_3_O_8_ and UO_2_ based on X‐ray diffraction data collected after heating U_3_O_8_ to 1173 K in air and subsequent cooling to room temperature (*R*_wp_  =  3.02%, GoF  =  1.15). Bragg markers denote from top to bottom: U_3_O_8_ (*Amm*2, *R*_Bragg_  =  0.707%, *a*  =  4.14618(9) Å, *b*  =  11.8903(3) Å, *c*  =  6.7426(2) Å, 97.9(1) wt.%), UO_2_ ($Fm\bar{3}m$, *R*_Bragg_  =  0.739%, a  =  5.433(2) Å, 2.1(1) wt.%).

Finally, U_3_O_8_ is retrieved with small amounts of UO_2+*x*_ left (Figure 6). The lattice parameters, however, differ significantly from the starting material (*a*  =  4.14618(9) Å, *b*  =  11.8903(3) Å, *c*  =  6.7426(2) Å as compared to *a*  =  4.14842(5) Å, *b*  =  11.9602(2) Å, *c*  =  6.7212(1) Å in the starting material). This discrepancy likely stems from different U/O ratios, also seen from the residual amount of UO_2+_ *_x_* in the phase mixture. The lattice parameter of the UO_2+*x*_ phase is significantly lower than for stoichiometric UO_2_ (*a*  =  5.433(2) Å as compared to *a*  =  5.4706 Å^[^ ^18^ ^]^) and even for UO_2.25_ (U_4_O_9_) suggesting an even higher oxygen content.^[^ ^18^ ^]^

A Rietveld refinement of the tetragonal structures of U_3_O_7_ (UO_2.33_) on this data, however was not suitable, neither were other ordered phases of lowered symmetry compared to the fluorite structure, like the orthorhombic structure of U_2_O_5_ (UO_2.5_).^[^ ^30^, ^35^, ^36^ ^]^ Due to its small share, a more detailed analysis and unambiguous identification of the phase was not possible. However, the lattice parameter indicates that the phase is a hyper‐stoichiometric fluorite‐related phase.

**Figure 7:**
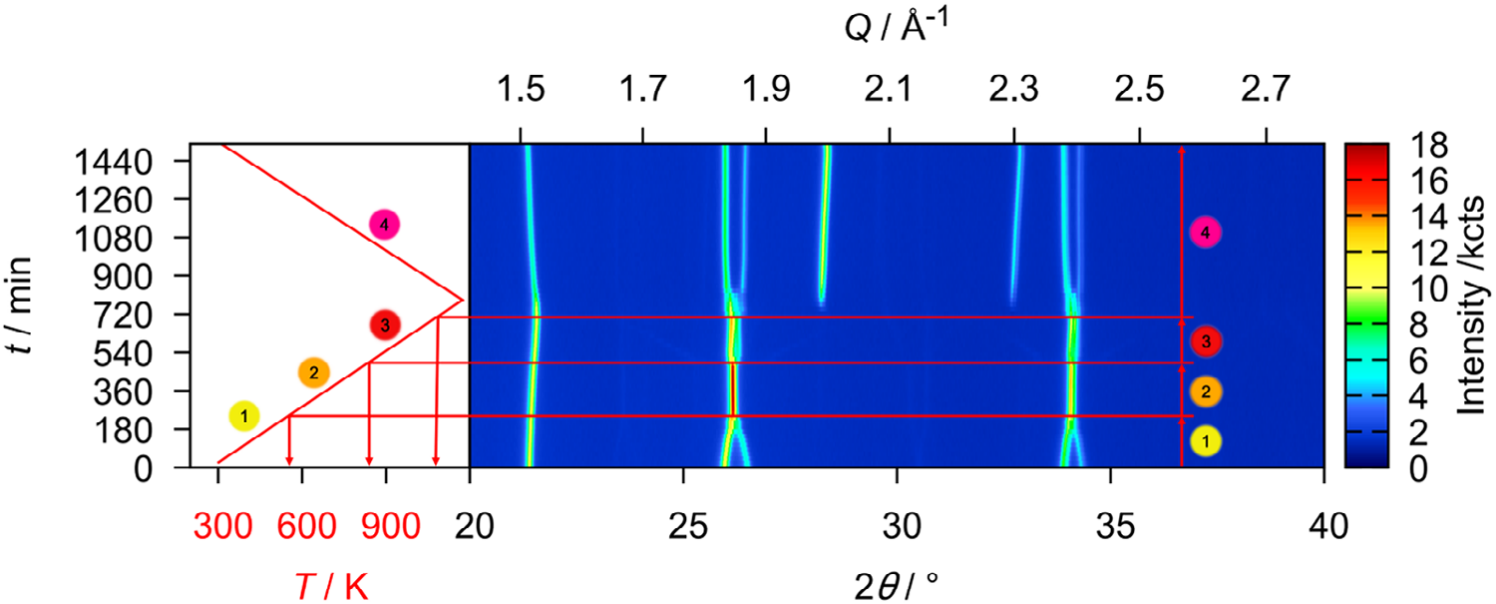
False‐color plot of part of the *in situ* XRPD (${\text{Cu-K}}_{\bar{\alpha }}$ radiation, BB geometry) data of U_3_O_8_ between room temperature and 1073 K in a static atmosphere of helium. During the heating period, three phases of the reaction can be observed: 1. Until 572 K the orthorhombic phase gradually approaches hexagonal symmetry. 2. Upon further heating the hexagonal phase thermally expands. 3. At 872 K, the formerly fused reflections suddenly split again, indicating a phase change likely caused by the beginning loss of oxygen. 4. The splitting of the reflections becomes more pronounced at 1122 K as a second phase appears which can be explained by a fluorite‐type phase UO_2+*x*_.

For comparison, a sample of U_3_O_8_ was heated under a static atmosphere of helium (*p*  = 1.3 bar), applying the same temperature program as for the measurement described before in air. The result of this procedure is shown in Figure 7. In the beginning of the experiment, the observations are identical to the ones described before heating the substance in air. The transition to hexagonal symmetry is completed at the same temperature of 572 K. As expected, the subsequent splitting of the reflections begins about 125 K earlier at 872 K, because of the absence of an oxygen partial pressure in the atmosphere, the formation of the phase named UO_2+*x*_ is observed from 1122 K on. In contrast to the measurement under air, the partial pressure of oxygen created from the oxygen loss from U_3_O_8_ is not sufficient to fully retrieve the starting material, such that a phase mixture remains in this case.

**Figure 8:**
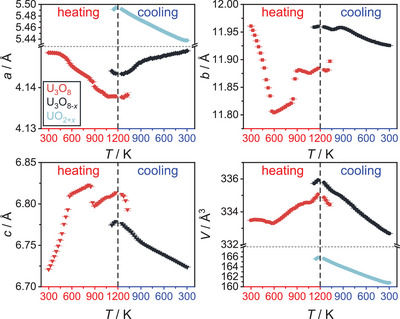
Evolution of the lattice parameters and the unit cell volume of all phases observed in the *in situ* X‐ray diffraction investigation of U_3_O_8_ in helium for temperatures up to 1173 K. The red symbols denote the evolution of U_3_O_8_, the black symbols denote a second phase of U_3_O_8_ structure found to exist simultaneously in this temperature range (U_3_O_8‐*x*_), the blue symbols denote a fluorite‐type phase UO_2+*x*_.

**Figure 9:**
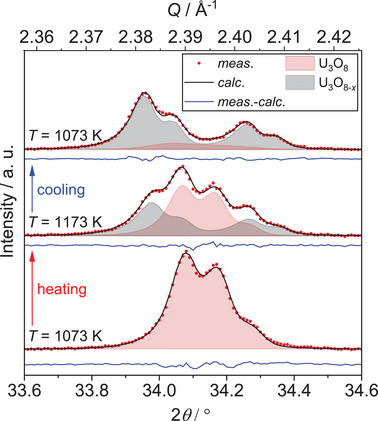
Selected Rietveld refinements based on XRPD data around the 131 and 102 reflections of U_3_O_8_ heating the substance from 1073 K to 1173 K and cooling back to 1073 K in a helium atmosphere. The temperature changes in steps of 100 K between the measurements. The area filled black indicates the corresponding reflections of a second U_3_O_8_‐type phase, showing its formation while the starting phase of U_3_O_8_ vanishes.

**Figure 10:**
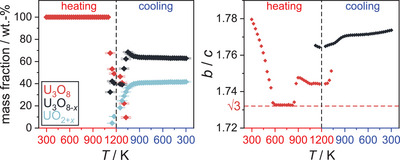
Evolution of the mass fractions of all phases observed in the *in situ* X‐ray diffraction experiment of U_3_O_8_ under a helium atmosphere.

From a sequential Rietveld refinement of the data the evolution of the lattice parameters of all phases was extracted (Figure 8). During the heating period, their behavior is very similar to the ones discussed before, except for the shift of the beginning of the oxygen loss to a lower temperature. The biggest difference is the preservation of the phase mixture observed as an intermediate state under air until the end of the measurement. The formation of the phase called U_3_O_8‐*x*_ can clearly be seen while the original phase of U_3_O_8_ completely vanishes (Figure 9). The evolution of the mass fractions of all phases shows their retention until room temperature is reached (Figure 10).

**Figure 11:**
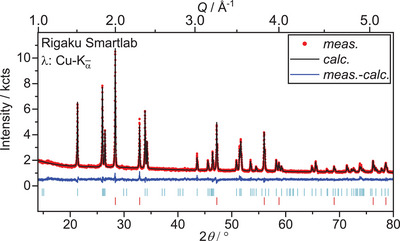
Rietveld refinement of the crystal structures of U_3_O_8_ and UO_2_ based on X‐ray diffraction data collected after heating U_3_O_8_ to 1173 K in an atmosphere of helium and subsequent cooling to room temperature (*R*_wp_  =  3.19%, GoF  =  1.22). Bragg markers denote from top to bottom: U_3_O_8‐*x*_ (*Amm*2, *R*_Bragg_  =  1.259%, *a*  =  4.14906(6) Å, *b*  =  11.9257(2) Å, *c*  =  6.7238(1) Å, 59.9(5) wt.%), UO_2_ ($Fm\bar{3}m$, *R*_Bragg_  =  2.341%, *a*  =  5.43804(5) Å, 40.1(5) wt.%).

A Rietveld analysis of the product mixture yields smaller lattice parameters for the U_3_O_8‐_ *_x_* phase compared to the product that is yielded under air (Figure 11). The lattice constant of the fluorite‐type phase is again conspicuously low, though larger than for the product in air. The larger phase fraction allows for a better identification of the phase, but still the ideal fluorite structure is the most suitable model to explain this diffraction pattern. There are no clear hints to whether this phase could be one of the other hyper‐stoichiometric oxides, that appear in the stoichiometric range between UO_2_ and U_3_O_8_ (UO_2.66_), which is why this phase is also referred to as UO_2+*x*_.

**Figure 12:**
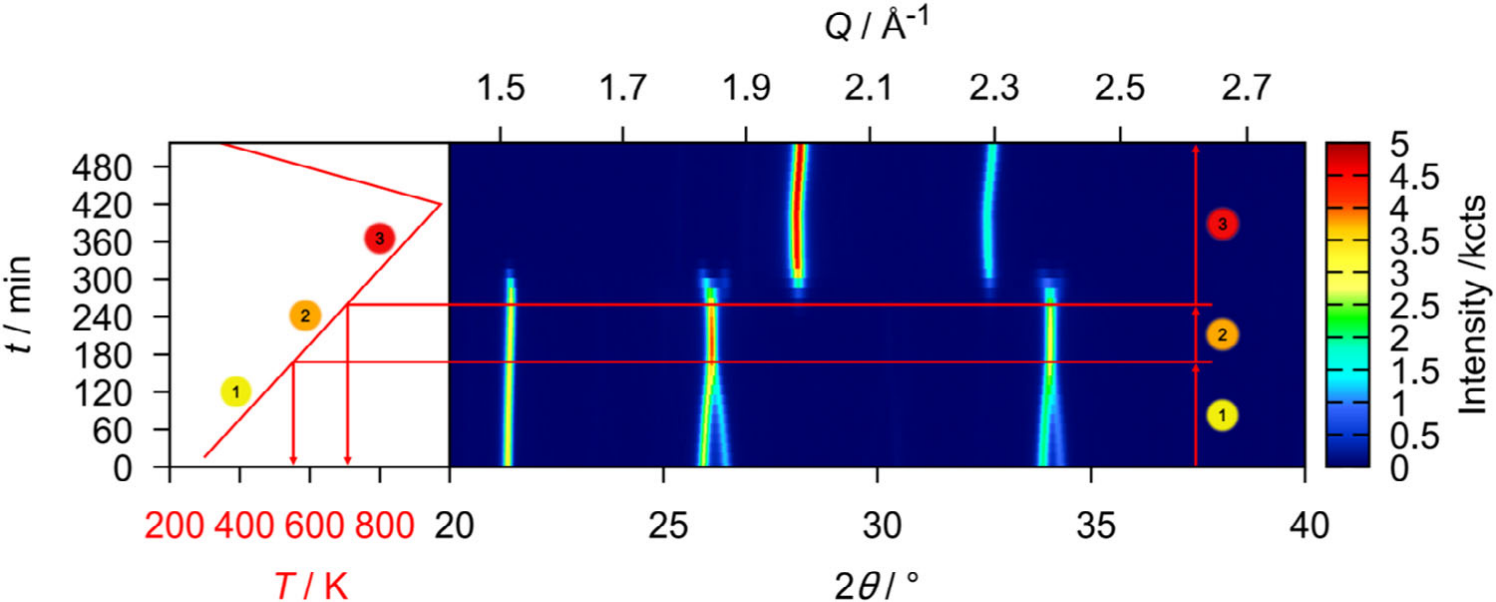
False‐color plot of part of the *in situ* XRPD (${\text{Cu-K}}_{\bar{\alpha }}$ radiation, PB geometry) data of U_3_O_8_ between room temperature and 973 K in a flow of 10 sccm of hydrogen under a pressure of 1.0 bar. During the reaction period, three phases of the reaction can be observed: 1. Until 622 K the orthorhombic phase gradually approaches hexagonal symmetry. 2. Upon further heating the hexagonal phase thermally expands. 3. At 722 K, the formerly fused reflections suddenly split again, indicating the start of the reaction with the simultaneous rapid formation of UO_2_, which is yielded as the final product at 847 K.

To investigate the behavior of U_3_O_8_ toward different reducing agents, first, a sample of U_3_O_8_ was exposed to a stream of hydrogen gas flowing through the powdered sample, applying a flow rate of 10 sccm and a pressure of 1.0 bar. It was then heated to a maximum temperature of 973 K at a rate of 30 Kmin^−1^, isothermally measuring a diffraction pattern every 50 K. It was then cooled with a rate of 30 Kmin^−1^ and a diffractogram was recorded every 100 K. The exposure time of each diffraction pattern was 8 minutes, scanning over a range of 10 – 90°. An overview plot of the result of this measurement is depicted in Figure 12.

**Figure 13:**
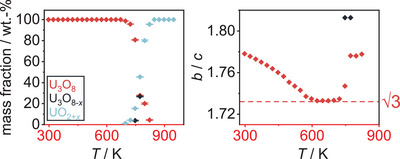
Evolution of the mass fractions of all phases observed in the *in situ* X‐ray diffraction experiment of the reduction of U_3_O_8_ by hydrogen gas.

**Figure 14:**
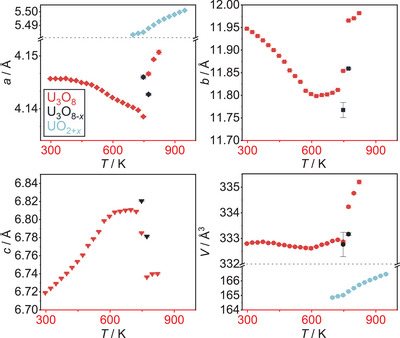
Evolution of the lattice parameters and the unit cell volume of all phases observed in the *in situ* X‐ray diffraction investigation of the reduction of U_3_O_8_ in hydrogen for temperatures up to 973 K.

In contrast to the former measurements, it can be seen that the U_3_O_8_ is herein fully transformed into a fluorite‐type phase. In the first part of the reaction, the phase transformation from orthorhombic to hexagonal U_3_O_8_ is again observed and completes at a temperature of 622 K, which is around 50 K later than for measurements in air or helium. Because a much larger overall heating rate was applied due to larger temperature steps between measurements and shorter exposure times this likely stems from an influence of the heating rate on the phase transformation. The starting point of the reaction was placed at 722 K, where a splitting of the hexagonal phase's reflection is first observed accompanied by the formation of a fluorite‐type phase. This phase quickly evolves and becomes the pure product of this reaction at 847 K as can be seen from the development of the mass fractions from Rietveld analysis of these data (Figure 13). The observations correspond well to earlier investigations on this reaction from literature, stating the formation of an oxygen‐deficient U_3_O_8_ phase, that returns to orthorhombic symmetry after its transformation to hexagonal.^[^ ^9^ ^]^ However, we find, that at the beginning of the reaction two phases of orthorhombic U_3_O_8_ structure exist simultaneously, with one possessing significantly deviating lattice parameters, just as observed during the measurements in air and under helium (Figure 14). Under the conditions investigated herein, U_3_O_8_ does though not seem to homogeneously react as proposed in the way that it continuously loses oxygen and returns to orthorhombic symmetry, but another intermediate phase of similar structure seems to play a role. To refer to the measurements described earlier, we will continue to call this phase U_3_O_8‐*x*_, since it also depicts an intermediate between the original U_3_O_8_ and a fluorite‐type phase UO_2+*x*_. In contrast to the reaction in air and helium, both U_3_O_8_ and U_3_O_8‐*x*_ seem to lose oxygen as indicated by the suddenly changing lattice parameters.

The product of the reaction was investigated by XRPD at room temperature. The diffraction pattern is very well described using the ideal fluorite‐type structure of UO_2_. The lattice parameter deviates much less from the one expected for stoichiometric UO_2_ (*a*  =  5.46514(9) Å as compared to 5.4702 Å), such that it can be identified as the expected product UO_2+*x*_ (*x*  =  0.034 – 0.072).^[^ ^18^ ^]^ This shows that the reaction remains incomplete and for a full conversion to stoichiometric UO_2_ the product would have had to be held at the maximum temperature of this experiment for a longer period of time.

**Figure 15:**
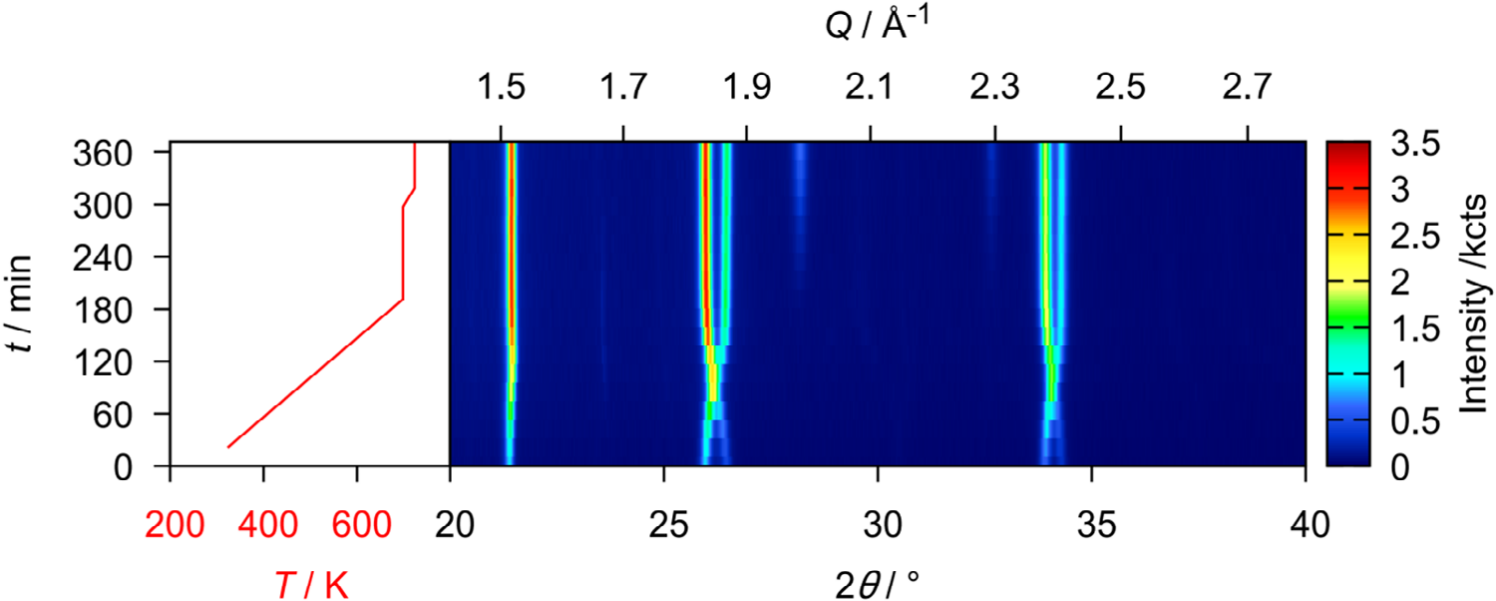
False‐color plot of part of the *in situ* XRPD (${\text{Cu-K}}_{\bar{\alpha }}$ radiation, PB geometry) data of U_3_O_8_ between room temperature and 723 K in a flow of 10 sccm of an argon hydrogen mixture (5 vol.% hydrogen) under a pressure of 1.3 bar.

To investigate the course of the reduction at a lower chemical potential of hydrogen, a sample of U_3_O_8_ was exposed to a mixture of 5 vol.% hydrogen in argon, applying a pressure of 1.3 bar and a flow rate of 10 sccm. It was then heated to 698 K, shortly before the reaction began in the measurement with pure hydrogen gas and held there for several measurements. In a second step the temperature was raised by another 50 K and held there for three measurements until the sample was quickly cooled to room temperature in a single step applying a cooling rate of 200 Kmin^−1^ (Figure 15).

**Figure 16:**
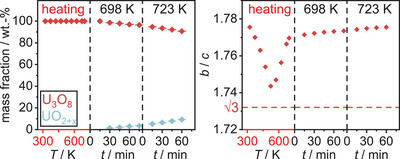
Evolution of the *b*/*c* ratio of U_3_O_8_ in the course of its partial reduction as observed in the *in situ* X‐ray diffraction experiment using a mixture of argon and 5 vol.% hydrogen. The value of √3 marks the ideal ratio at which the structure adopts a hexagonal symmetry.

**Figure 17:**
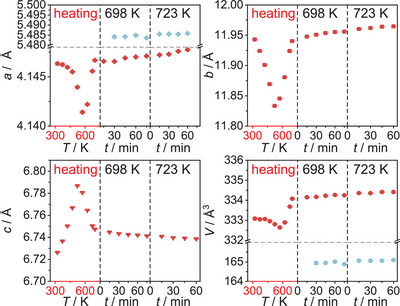
Evolution of the lattice parameters and the unit cell volume of all phases observed in the *in situ* X‐ray diffraction investigation of the reduction of U_3_O_8_ in a mixture of argon and 5 vol.‐% hydrogen for temperatures up to 723 K.

**Figure 18:**
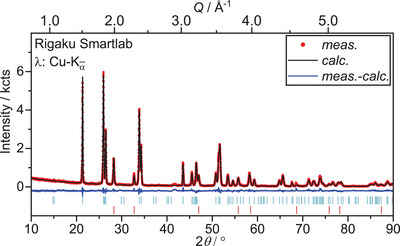
Rietveld refinement of the crystal structures of U_3_O_8_ and UO_2_ based on X‐ray diffraction data collected after heating U_3_O_8_ to 723 K in an atmosphere of 5 vol.% hydrogen in argon and rapid cooling to room temperature (*R*_wp_  =  9.50%, GoF  =  1.46). Bragg markers denote from top to bottom: U_3_O_8‐*x*_ (*Amm*2, *R*_Bragg_  =  2.741%, *a*  =  4.15109(8) Å, *b*  =  11.9439(2) Å, *c*  =  6.7183(1) Å, 87.69(19) wt.%), UO_2_ ($Fm\bar{3}m$, *R*_Bragg_  =  3.852%, a  =  5.4624(1) Å, 12.3(2) wt.%).

In contrast to the reaction with pure hydrogen, the phase transition observed at the beginning does not take place in this case, as the *b*/*c* ratio does not reach the value of √3 (Figure 16). Instead, it goes through a minimum value. Particularly noteworthy is the reversal of the lattice parameter trend for the *b* and *c* parameter in comparison to pure hydrogen: Whereas there the *b* parameter decreases and the *c* parameter increases upon start of the reaction, here they behave vice versa, resembling the course of the lattice parameters of the phase U_3_O_8‐*x*_ intermediately formed upon reduction with pure hydrogen (Figure 17). No such phase is observed in this experiment. This leads to the conclusion that in this case, due to the slower overall heating rate and the smaller amount of hydrogen available, U_3_O_8_ seems to be continuously transformed into the intermediate phase observed before. The substance continues to slowly being reduced during the two dwelling periods, but as expected the reaction proceeds much slower in this case. To retrieve and further examine the phase, the reaction was aborted, and the product quickly cooled down to room temperature, where it was investigated by XRPD (Figure 18).

Rietveld refinement of the crystal structures of orthorhombic U_3_O_8_ and fluorite‐type UO_2_ on this data yielded a good explanation of the measured diffraction pattern. The lattice parameters of U_3_O_8_ resemble the ones refined for the starting material (*a*  =  4.15109(8) Å, *b*  =  11.9439(2) Å, *c*  =  6.7183(1) Å as compared to *a*  =  4.14622(6) Å, *b*  =  11.9542(2) Å, *c*  =  6.7189(1) Å in the starting material), such that it can be identified as a slightly oxygen‐deficient phase of U_3_O_8_ (U_3_O_8‐*x*_).

Based on the lattice parameter of the secondary UO_2+_ *_x_* phase, the composition is estimated to be UO_2.088_, showing a higher oxygen excess than before, in agreement with the lower turnover of the reaction.^[^ ^18^ ^]^ By slow conduction of the reduction reaction, the process becomes continuous, and the reaction proceeds such that only one phase of U_3_O_8_ is observed throughout the course of the reaction. These findings therefore confirm the findings described before in literature and as expected show that the partial pressure of hydrogen has an influence on the reaction on the time scale of this *in situ* X‐ray diffraction experiment.

**Figure 19:**
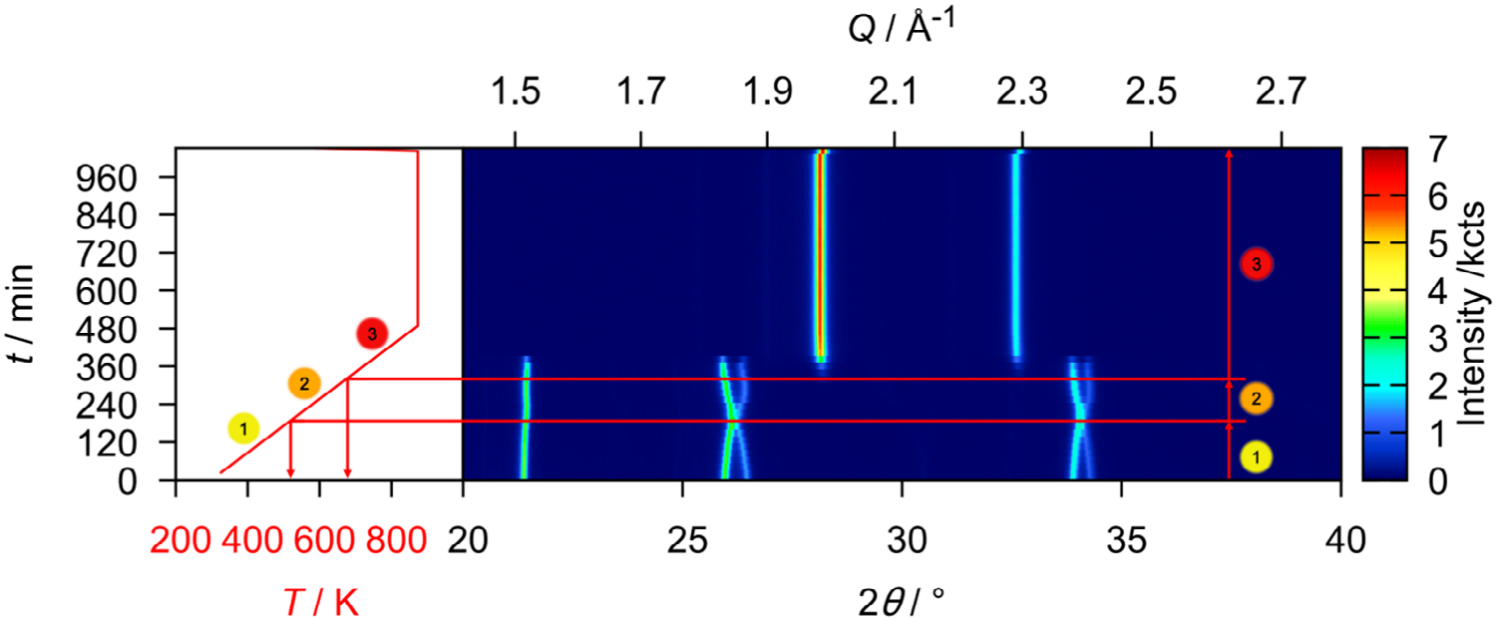
False‐color plot of part of the *in situ* XRPD (${\text{Cu-K}}_{\bar{\alpha }}$ radiation, PB geometry) data of a mixture of U_3_O_8_ and PVDF between room temperature and 873 K in a dynamic vacuum. During the reaction period, three phases of the reaction can be observed: 1. Until 522 K the orthorhombic phase gradually approaches hexagonal symmetry, without completing the phase transition. 2. Upon further heating the reflections split again. 3. At 697 K the rapid formation of UO_2_ begins, which is yielded phase pure as the final product at 797 K.

In the course of our studies on heteroanionic uranium oxides, we performed several preliminary reactions of U_3_O_8_ and polyvinylidene fluoride (PVDF) known to be able to fluorinate rare‐earth oxides to form fluoride oxides.^[^ ^21^, ^37^ ^]^ In our case however, all reactions resulted in the reduction of U_3_O_8_ to UO_2_. To see whether heteroanionic intermediates are formed in the course of this reaction and if the reaction pathway differed from the one observed for hydrogen, U_3_O_8_ and, PVDF were ground under *n*‐pentane in a stoichiometric ratio of 1:1 until the *n*‐pentane had completely evaporated. This reaction mixture was then exposed to a dynamic vacuum and heated to a temperature of 873 K and held there for about 7 hours. During this temperature program, X‐ray diffraction patterns were recorded *in situ* every 25 K with an exposure time of 14 minutes covering an angular range of 10–80° (Figure 19).

**Figure 20:**
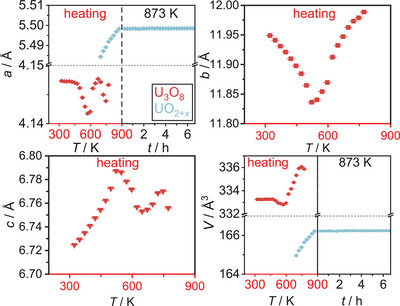
Evolution of the lattice parameters and the unit cell volume of all phases observed in the *in situ* X‐ray diffraction investigation of the reaction of U_3_O_8_ with PVDF in a dynamic vacuum for temperatures up to 873 K.

**Figure 21:**
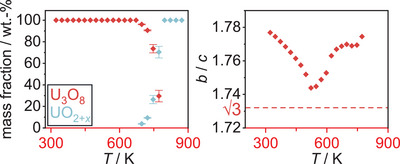
Left: Evolution of the mass fractions of all phases observed during the reaction of U_3_O_8_ with PVDF. Right: Evolution of the *b*/*c* ratio of U_3_O_8_ in the course of its reaction with PVDF as observed in the *in situ* X‐ray diffraction experiment under dynamic vacuum. The value of √3 marks the ratio at which the structure adopts an ideally hexagonal symmetry.

Again, the complete conversion of the starting material U_3_O_8_ to UO_2+_ *_x_* is observed. The lattice parameter of the product by Rietveld refinement of the crystal structure based on the room temperature diffraction data yields *a*  =  5.46561(9) Å, a value very similar to the one obtained from the product of the reduction of U_3_O_8_ by hydrogen and therefore proposing a very similar oxygen content of this product (UO_2.072_–UO_2.034_). The evolution of the lattice parameters of the phases (Figure 20) first shows the proceeding transformation toward the hexagonal phase of U_3_O_8_ as seen for all of the formerly described experiments. In this reaction, the *b*/*c* ratio for an ideal hexagonal symmetry is not reached as already observed in the reaction with the argon‐hydrogen mixture (Figure 21). The minimum of the *b*/*c* ratio at 522 K is therefore determined as the starting point of the reaction. From the observations of the lattice parameter evolution, the development until the start of the reaction is very similar to the slow reduction using the argon‐hydrogen mixture described before.

A conspicuous difference occurs as soon as the formation of UO_2+_ *_x_* begins to be observable by X‐ray diffraction at 697 K. Here the trend in lattice parameter evolution of the *a* and *c* lattice parameters reverses and *a* starts to decrease, while *c* increases again, such that at 747 K, *a* shows a second minimum value and *c* a second maximum value. In the experiments before, such behavior was not observed, hinting toward a different pathway or mechanism of the reduction by this reducing agent. Under the conditions investigated in this experiment, however, no heteroanionic compound could be identified, for example by possessing a structure that would be distinguishable from U_3_O_8‐*x*_ or UO_2+*x*_ by X‐ray diffraction. Possibly, a solid solution with mixed anions is forming intermediately, it could, however not be isolated in the course of these investigations. Additionally, there are no reports on such phases in literature yet, making it hard to compare lattice parameter deviations from the data acquired herein. Experiments trying to dissolve the product in diluted acids for fluorine determination by a fluoride‐sensitive electrode failed as no significant dissolution of the product could be observed.

**Figure 22:**
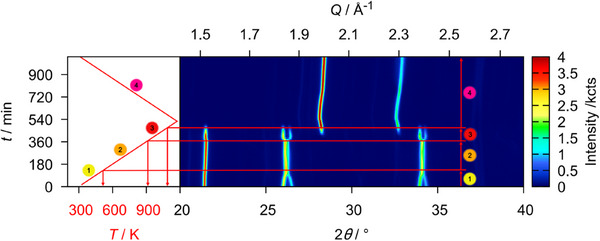
False‐color plot of part of the *in situ* XRPD (${\text{Cu-K}}_{\bar{\alpha }}$ radiation, PB geometry) data of U_3_O_8_ between room temperature and 1173 K in a dynamic vacuum. During the reaction period, three phases of the reaction can be observed: 1. Until 548 K the orthorhombic phase gradually approaches hexagonal symmetry, without completing the phase transition. 2. Upon further heating the reflections split again. 3. At around 900 K the rapid formation of UO_2+*x*_ begins, which is yielded as the final product at 1197 K.

In order to compare the behavior in the presence and absence of PVDF, a reference experiment observing the behavior of U_3_O_8_ in a similar dynamic vacuum was performed. Therefore, the sample was heated to 1173 K in a dynamic vacuum measuring an XRPD pattern in the angular range of 10 – 90° every 25 K, in between which a heating rate of 30 Kmin^−1^ was applied. The resulting evolution of the corresponding XRPD patterns can be found in Figure 22.

**Figure 23:**
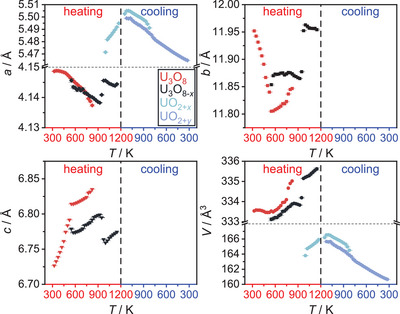
Evolution of the lattice parameters and the unit cell volume of all phases observed in the *in situ* X‐ray diffraction investigation of U_3_O_8_ in a dynamic vacuum for temperatures up to 1173 K.

**Figure 24:**
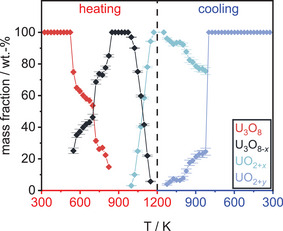
Evolution of the mass fractions of all phases observed during the reaction of U_3_O_8_ in a dynamic vacuum. The symbols were connected as a guide for the eye.

As in the case of U_3_O_8_ and PVDF, a fluorite‐type phase is formed during this reaction. This especially contrasts the behavior of U_3_O_8_ in air or under an inert gas atmosphere, as in these cases, this product reacts back to a U_3_O_8_ type phase or the reaction remains incomplete (Figure 2 and Figure 7). The starting temperature of the transformation, however, is in good agreement with the ones observed for aerobic conditions and under helium. As expected, the reduced pressure drives the phase toward this product. Compared to the reaction with PVDF, three major differences can be observed: First, the transformation proceeds via the intermediate U_3_O_8‐_ *_x_* phase in the case of pure U_3_O_8_, which is not observed as a phase refined separately from U_3_O_8_ in the reaction with PVDF. This might be due to the reaction starting before the formation temperature of this second phase in dynamic vacuum, quickly transforming the almost hexagonal U_3_O_8_ to the more orthorhombic oxygen‐deficient phase, such that the two phases do not appear simultaneously in any of the measurements. Second, the reaction to form UO_2+_ *_x_* begins at a temperature around 200 K lower when reacting U_3_O_8_ with PVDF, excluding a pure oxygen emission caused by the reduced pressure in the reaction with PVDF. In the measurement without PVDF the temperature range in which the phase U_3_O_8‐_ *_x_* is observed is also rather long. This is different as for the air or helium atmospheres, where the separately refined phase U_3_O_8‐_ *_x_* is formed at more elevated temperatures, while under dynamic vacuum this phase appears at temperatures as early as 548 K, from when on it becomes the principal phase. The refinement of the exact structure is however hampered by the appearance of several low‐intensity reflections, making the fit by the structure model of U_3_O_8_ rather questionable and hinting toward a more complex situation in this temperature region, similar was explained in the measurements before. The exact determination of the structural composition of these phases would, however, exceed the scope of this study though it represents an interesting finding for possible future investigations. The last difference of this measurement compared to the reaction with PVDF is that the product seems to be a mixture of UO_2+*x*_‐phases with different values of *x* (named UO_2+*x*_ and UO_2+*y*_ with *x* ≠ *y* in Figure 23, 24). In the sequential refinement of the *in situ* data, another phase was observed near the end of the cooling period and in the ultimate product. None of the known uranium oxide phases accounts for the additional reflections observed in the product. Additionally, the diffraction pattern was only incompletely explained by the use of UO_2+*x*_ as a structural model, hinting toward the product of this reaction being substantially different from the fluorite type or containing another unknown phase and the reaction with PVDF forming a completely different product. This is supported by the much smaller lattice parameter of this product (*a*  =  5.4351(1) Å) compared to the reaction product with PVDF (*a*  =  5.46561(9) Å).

**Figure 25:**
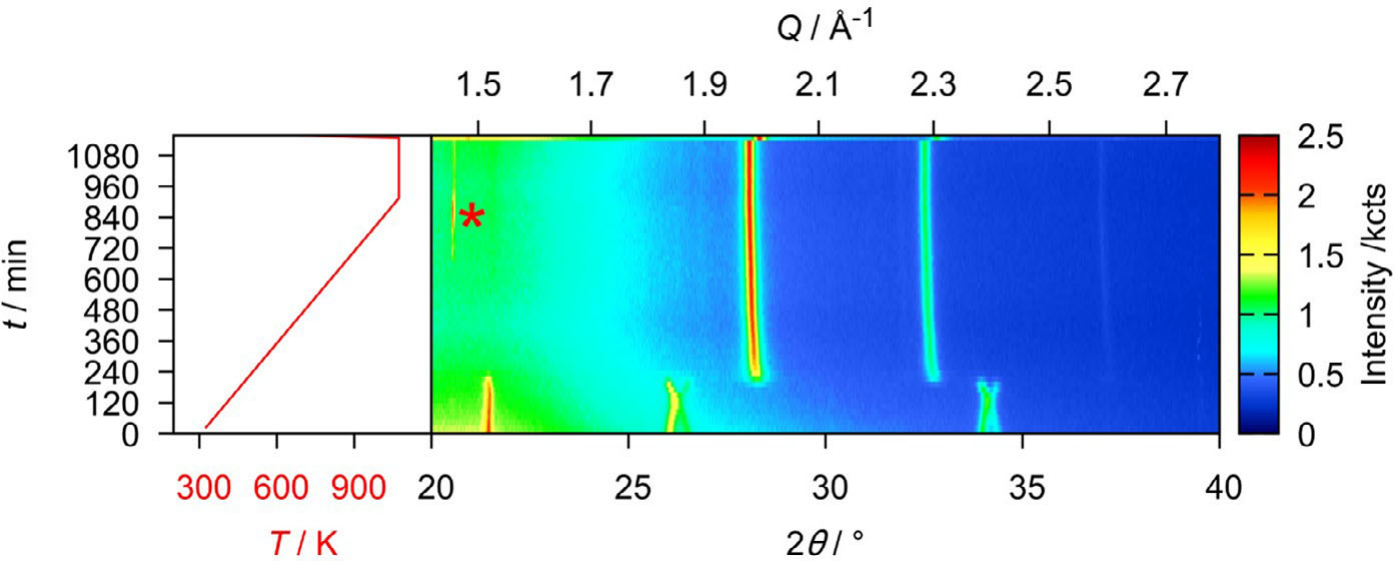
False‐color plot of the *in situ* XRPD (${\text{Cu-K}}_{\bar{\alpha }}$ radiation, PB geometry) data of a mixture of U_3_O_8_ and CaH_2_ (1:2 stoichiometric ratio) between room temperature and 1073 K in a silica glass capillary under an argon atmosphere. The intensity marked with an asterisk (*) is an artifact of the measurement and does not correspond to any phase observed in the experiment.

**Figure 26:**
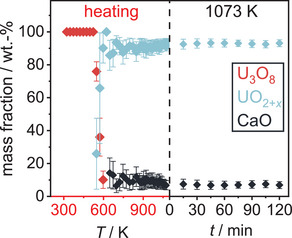
Evolution of the mass fractions of all phases observed in the *in situ* X‐ray diffraction experiment of the reaction of U_3_O_8_ and CaH_2_.

Finally, U_3_O_8_ was reacted with CaH_2_ in order to see whether heteroanionic hydrides would form as intermediates in the course of this reaction. To do so, a mixture of U_3_O_8_ and CaH_2_ in the stoichiometric ratio 1:2 was thoroughly ground and mixed with about twice the volume of diamond powder in order to reduce its X‐ray absorption during the diffraction measurement. This mixture was placed in a graphitized silica glass capillary that was sealed under an argon atmosphere. The sample was first heated to 873 K applying a heating rate of 30 Kmin^−1^ and measuring a diffraction pattern in the angular region of 20–40° every 25 K. After that the sample was brought to a temperature of 1073 K using the same heating rate, but conducting a measurement every 10 K. It was dwelled at this temperature for 2 h before cooling it to room temperature with a heating rate of 50 Kmin^−1^. The result of this experiment is depicted in Figure 25. Due to the higher absorption of the sample in transmission measurements and an enlarged background due to the scattering of the silica glass capillary itself, the data quality is much poorer compared to the flat specimen measurements discussed before, however the development of the phase composition as well as lattice parameters remained well determined, though possessing a larger relative error. No reflections of the CaH_2_ were observed, probably due to higher background and comparatively low intensities. The overview plot of the diffraction experiment shows a typical reduction process as seen in most of the experiments described before following the equation:

$$U_{3}O_{8}+2\;\mathrm{Ca}H_{2}\to 3\;UO_{2}+2\;\mathrm{CaO}+2H_{2}$$

and the temperature of 472 K at the minimum value of the *b*/*c* ratio is declared the starting point of the reaction, after which the deviation from hexagonal symmetry becomes more pronounced due to the beginning loss of oxygen. After that, the reaction is completed very quickly in the course of three measurements (Figure 26) leaving UO_2+*x*_ and CaO as the product mixture. Rietveld refinement of their structures on the diffraction pattern at room temperature yielded a lattice parameter of *a*  =  5.464(2) Å for UO_2+*x*_, which estimates the oxygen content to UO_2.174_–UO_2.123_ which is rather high compared to former experiments using different reducing agents. The phase transition of U_3_O_8_ is again well observed. In this case the ideal hexagonal symmetry is once again not reached.

According to the chemical reaction equation (see above) the product should be stoichiometric UO_2_ with a molar ratio of 3:2 with respect to CaO. Rietveld analysis yields 73(1) mol.% UO_2_ and 27(1) mol.% CaO, which could be due to the large difference in molar masses of the starting materials, such that the weighing errors are not negligible. Moreover, the error in the quantitative determination by Rietveld analysis is considerable due to X‐ray absorption, resulting in a relatively high error in the resulting stoichiometric composition. Apart from these sources of systematic error it is shown that among the reactions investigated herein, this reaction proceeds the fastest. No indication of intermediately formed phases other than substoichiometric U_3_O_8‐*x*_ are observed. The lattice parameter of the CaO formed (*a*  =  4.814(2) Å) corresponds well to literature values.^[^ ^38^ ^]^

The conditions investigated and products of all the reductions investigated herein are summarized in Table 2. The heating rates therein were averaged accounting for the different exposure times and temperature differences between measurements. Clearly under these conditions, the solid‐solid reactions lower the starting temperature of the reduction significantly compared to the solid‐gas reactions. However, the temperature difference between the start of the reaction and complete turnover to UO_2+*x*_ remains smallest for the reduction by hydrogen gas, possibly also due to the relatively high heating rate applied. A big difference from the solid‐solid reductions is also the unlimited amount of reducing agent for the solid‐gas reactions, which leads to a continuous lowering of the oxygen content of UO_2+*x*_. In contrast, the amount of reducing agent is limited for the solid‐solid reduction reactions, which makes it more likely that the reduction is completed after complete turnover is detected, for example by an invariance of the phase share of CaO in the reduction by CaH_2_.

**Table 2:** Summary of the investigated conditions for the reduction of U_3_O_8_, average heating rates, characteristic temperatures, and lattice parameters of the product phases UO_2+*x*_.

Conditions	Δ*T*_av_ / Kmin^−1^	*T*_start_ / K	*T*_end_ / K	*a* (UO_2+_ *_x_*) / Å
**Air (ambient pressure)**	1.57	997	‐	5.433(2)
**He (1.3 bar)**	1.57	872	‐	5.43804(5)
**H_2_ (1.0 bar, 10 sccm)**	5.17	722	847	5.46518(9)
**Ar + 5 vol.% H_2_ (1.0 bar, 10 sccm)**	2.83	572	‐	5.4624(1)
**PVDF (1 eq., dyn. vacuum)**	1.69	522	797	5.46561(9)
**Dynamic vacuum**	1.57	973	1173	5.4351(1)
**CaH_2_ (2 eq.)**	1.81 0.72	472	622	5.464(2)

## 4 Conclusion

This study provides insights into the reduction processes of U_3_O_8_ by various reducing agents through the application of continuous *in situ* X‐ray diffraction. The findings highlight the complexity, especially regarding nonstoichiometry, and the intermediate phases involved in the reduction process, offering a more detailed understanding of the reaction pathways and structural transitions of uranium oxides under different conditions.

Under air and helium, U_3_O_8_ transitions from its orthorhombic structure to a hexagonal modification at around 572 K, by anisotropic changes in lattice parameters. Further heating leads to oxygen loss and the formation of an oxygen‐rich fluorite‐type UO_2+_ *_x_* phase. In a helium atmosphere, this transition is accompanied by the emergence of a phase of U_3_O_8_‐structure possessing significantly different lattice parameters (U_3_O_8‐*x*_), which in the case of heating in air is only formed intermediately, suggesting a reversible oxidation‐reduction process at elevated temperatures. The lattice parameters of the UO_2+_ *_x_* phases produced in these reactions are significantly lower than is expected for stoichiometric UO_2_ and compared to the reactions with reducing agents investigated herein, however no hint toward a hyper‐stoichiometric ordered variant (U_4_O_9_, U_3_O_7_) was observed in the diffraction patterns.

The reduction by hydrogen gas follows a distinct pathway where U_3_O_8_ fully transforms into a fluorite‐type UO_2+*x*_ phase at a temperature of 847 K. The phase transition to hexagonal U_3_O_8_ occurs at a slightly higher temperature (622 K) in hydrogen, and the reaction proceeds through intermediate phases, including a simultaneous existence of two orthorhombic U_3_O_8_ phases. When reduced by a hydrogen‐argon mixture, the reduction of U_3_O_8_ proceeds more gradually, highlighting the impact of reduced hydrogen partial pressure and heating rates. The presence of only one orthorhombic U_3_O_8_ phase throughout the reaction suggests a continuous transformation without the formation of two phases of U_3_O_8_‐structure seen with pure hydrogen. Such small differences in the reaction path can have a decisive influence on the microstructure of powders. In technical processes, these in turn are important for many product properties.^[^ ^39^, ^40^ ^]^

Our preliminary investigations into the use of PVDF and CaH_2_ as reducing agents did not lead to the formation of heteroanionic uranium oxides but similarly resulted in the reduction of the oxide, mirroring the behavior observed with hydrogen. This indicates that the reducing environment predominantly influences the reaction pathway toward the formation of UO_2_ and its hyper‐stoichiometric variants (Supporting information).

Overall, the continuous *in situ* X‐ray diffraction technique has proven to be a powerful tool in elucidating the structural evolution and reaction pathways of U_3_O_8_ under varying reducing conditions. These findings provide valuable information on processes involved in uranium refinement and nuclear fuel production. Future work could explore more diverse reducing environments and further refine the understanding of intermediate phase formations, potentially leading to new insights into the stability and reactivity of uranium oxides.

## Conflict of Interest

The authors declare no conflict of interest.

## Supporting information



Supporting Information

## Data Availability

The data that support the findings of this study are available from the corresponding author upon reasonable request.
