# Supplementary material for: In Situ X‐ray Diffraction Studies on the Reduction of U3O8 by Various Reducing Agents
Source: Chemistry. 2025 May 27;31(34):e202500978. doi: 10.1002/chem.202500978 (PMC12172588; doi:10.1002/chem.202500978)
Supplement: Supplementary file 1 — Supporting Information [file CHEM-31-e202500978-s001.pdf]

Supplementary material

In situ X-ray Diffraction Studies on the Reduction of U_3O_8 by various Reducing Agents

Marvin Michak[a], Frank-Constantin Ideker[a], Holger Kohlmann[a*]

[a] Leipzig University, Faculty of Chemistry, Institute of Inorganic Chemistry and Crystallography, Johannisallee 29, 04103 Leipzig, Germany, *Email: holger.kohlmann@uni-leipzig.de, phone: +49 3419736201

Full-range false-color plots, tables of all refined lattice parameters and phase fractions and Rietveld refinements of the final products of the *in situ* X-ray diffraction experiments

1. U_3O_8 in Air

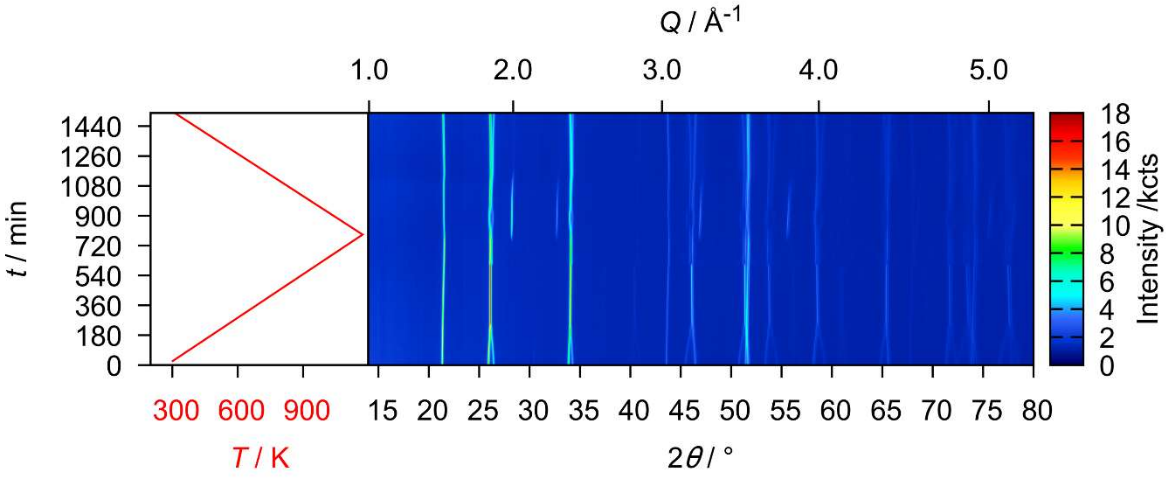

Figure S1: False-color plot of the *in situ* X-ray powder diffraction (Cu-$K_{\bar{\alpha}}$ radiation, BB geometry) data of U_3O_8 between room temperature and 1173 K at a static atmosphere of air.

Table S1: Refined lattice parameters and phase fractions of all phases appearing in the *in situ* X-ray diffraction experiment of U_3O_8 in air.

ID	*T* / K	phase	*a* / Å	*b* / Å	*c* / Å	*V* / Å^3	*ω* / wt.-%
**0**	298	U_3O_8	4.14844(5)	11.9607(2)	6.7211(1)	333.490(9)	100
**1**	322	U_3O_8	4.14863(5)	11.9516(2)	6.7269(1)	333.538(9)	100
**2**	347	U_3O_8	4.14853(5)	11.9434(2)	6.7315(1)	333.529(9)	100
**3**	372	U_3O_8	4.14852(5)	11.9327(2)	6.7374(1)	333.520(9)	100
**4**	397	U_3O_8	4.14840(5)	11.9215(2)	6.7438(1)	333.516(9)	100
**5**	422	U_3O_8	4.14836(5)	11.9099(2)	6.7504(1)	333.512(9)	100
**6**	447	U_3O_8	4.14821(5)	11.8972(2)	6.7577(1)	333.506(9)	100
**7**	472	U_3O_8	4.14783(6)	11.8816(2)	6.7663(1)	333.460(9)	100
**8**	497	U_3O_8	4.14755(5)	11.8604(2)	6.7777(1)	333.404(8)	100
**9**	522	U_3O_8	4.14718(6)	11.8461(2)	6.7860(1)	333.383(9)	100
**10**	523	U_3O_8	4.14717(6)	11.8464(2)	6.7862(1)	333.399(9)	100
**11**	547	U_3O_8	4.14664(5)	11.8305(2)	6.7955(1)	333.365(9)	100
**12**	572	U_3O_8	4.14578(4)	11.8043(7)	6.8107(4)	333.30(3)	100
**13**	597	U_3O_8	4.14540(4)	11.8037(5)	6.8129(4)	333.36(2)	100
**14**	622	U_3O_8	4.14492(4)	11.8070(4)	6.8125(3)	333.40(2)	100
**15**	647	U_3O_8	4.14454(4)	11.8080(5)	6.8140(3)	333.47(2)	100
**16**	672	U_3O_8	4.14425(4)	11.8125(2)	6.8142(1)	333.585(9)	100
**17**	697	U_3O_8	4.14389(4)	11.8140(2)	6.8153(1)	333.650(9)	100
**18**	722	U_3O_8	4.14367(4)	11.8162(2)	6.8165(1)	333.754(9)	100
**19**	747	U_3O_8	4.14335(4)	11.8185(2)	6.8177(1)	333.851(9)	100
**20**	772	U_3O_8	4.14299(4)	11.8202(2)	6.8190(1)	333.932(9)	100
**21**	797	U_3O_8	4.14278(4)	11.8222(2)	6.8204(1)	334.039(9)	100
**22**	822	U_3O_8	4.14252(4)	11.8235(3)	6.8221(1)	334.14(1)	100
**23**	847	U_3O_8	4.14230(4)	11.8262(2)	6.8232(1)	334.25(1)	100
**24**	872	U_3O_8	4.14217(4)	11.8284(3)	6.8250(1)	334.39(1)	100
**25**	897	U_3O_8	4.14197(4)	11.8306(3)	6.8266(2)	334.52(1)	100
**26**	922	U_3O_8	4.14180(4)	11.8331(3)	6.8280(2)	334.64(1)	100
**27**	947	U_3O_8	4.14163(4)	11.8353(3)	6.8295(2)	334.77(1)	100
**28**	972	U_3O_8	4.14146(4)	11.8381(3)	6.8308(1)	334.89(1)	100
**29**	997	U_3O_8	4.13900(6)	11.8854(1)	6.80410(8)	334.720(8)	100
**30**	1022	U_3O_8	4.13892(6)	11.8827(1)	6.80690(8)	334.774(7)	100
**31**	1047	U_3O_8	4.13848(6)	11.8804(1)	6.80857(8)	334.757(7)	100
**32**	1072	U_3O_8	4.13822(5)	11.8790(1)	6.81016(8)	334.774(7)	100
**33**	1097	U_3O_8	4.13776(5)	11.8784(1)	6.81062(7)	334.741(7)	100

**34**	1122	U_3O_8	4.13788(5)	11.8804(1)	6.81089(7)	334.822(7)	100
**35**	1147	UO_2	5.4837(6)			164.90(6)	2.6(1)
		U_3O_8	4.13773(6)	11.8829(1)	6.81172(8)	334.921(7)	97.4(1)
**36**	1172	UO_2	5.4945(1)			165.88(1)	8.8(2)
		U_3O_8	4.13783(6)	11.8854(2)	6.81349(9)	335.087(8)	91.2(2)
**37**	1149	U_3O_8	4.13780(6)	11.8831(2)	6.81137(9)	334.915(8)	83.2(3)
		UO_2	5.49245(7)			165.691(6)	16.8(3)
**38**	1124	UO_2	5.49006(6)			165.475(6)	22.2(4)
		U_3O_8	4.13770(7)	11.8819(2)	6.8086(1)	334.735(9)	77.8(4)
**39**	1099	UO_2	5.48847(6)			165.331(6)	25.1(4)
		U_3O_{8-x}	4.1432(2)	11.9541(4)	6.7734(3)	335.48(2)	21.7(5)
		U_3O_8	4.13836(8)	11.8816(2)	6.8066(1)	334.68(1)	53.2(5)
**40**	1074	U_3O_{8-x}	4.1434(1)	11.9535(3)	6.7722(2)	335.41(2)	34.7(5)
		U_3O_8	4.1388(1)	11.8843(3)	6.8019(2)	334.56(2)	39.6(5)
		UO_2	5.48643(6)			165.147(6)	25.7(4)
**41**	1049	U_3O_8	4.1394(2)	11.8871(4)	6.7978(3)	334.49(2)	29.1(5)
		U_3O_{8-x}	4.14363(9)	11.9525(2)	6.7704(2)	335.31(1)	44.6(6)
		UO_2	5.48430(6)			164.955(5)	26.3(4)
**42**	1024	U_3O_{8-x}	4.14371(8)	11.9518(2)	6.7683(1)	335.20(1)	46.6(6)
		U_3O_8	4.1394(2)	11.8843(5)	6.7956(3)	334.30(2)	27.3(5)
		UO_2	5.4821(6)			164.752(5)	26.1(4)
**43**	999	U_3O_8	4.1387(1)	11.8779(4)	6.7982(2)	334.19(2)	34.9(5)
		UO_2	5.48001(7)			164.567(6)	26.4(4)
		U_3O_{8-x}	4.14387(9)	11.9499(2)	6.7668(2)	335.08(1)	39(6)
**44**	974	U_3O_{8-x}	4.1433(1)	11.9437(4)	6.7661(2)	334.83(2)	24.7(5)
		U_3O_8	4.13810(9)	11.8725(2)	6.7990(1)	334.03(1)	50.2(6)
		UO_2	5.47782(7)			164.370(6)	25.2(4)
**45**	949	U_3O_8	4.13774(8)	11.8689(2)	6.7986(1)	333.88(1)	64.2(6)
		U_3O_{8-x}	4.1404(3)	11.9278(9)	6.7671(5)	334.20(4)	11.9(6)
		UO_2	5.47577(7)			164.186(7)	23.9(4)
**46**	924	UO_2	5.47406(7)			164.032(7)	22.4(3)
		U_3O_8	4.13761(7)	11.8665(2)	6.7980(1)	333.776(9)	71.4(5)
		U_3O_{8-x}	4.1403(4)	11.909(1)	6.7616(6)	333.40(6)	6.2(4)
**47**	899	U_3O_8	4.13777(6)	11.8664(2)	6.79603(9)	333.69(8)	79.3(3)
		UO_2	5.47227(8)			163.871(7)	20.7(3)
**48**	874	U_3O_8	4.13773(7)	11.8647(2)	6.79411(1)	333.544(8)	80.7(3)
		UO_2	5.47029(1)			163.694(9)	19.3(3)
**49**	849	U_3O_8	4.13762(7)	11.8635(2)	6.7921(1)	333.400(9)	81.9(3)
		UO_2	5.4682(1)			163.51(1)	18.1(3)
**50**	824	UO_2	5.4660(1)			163.31(1)	16.6(3)
		U_3O_8	4.13740(7)	11.8619(2)	6.78962(1)	333.216(9)	83.4(3)
**51**	799	UO_2	5.4638(2)			163.11(1)	14.9(3)
		U_3O_8	4.13741(7)	11.8610(2)	6.7875(1)	333.091(9)	85.1(3)
**52**	774	U_3O_8	4.13630(8)	11.8573(2)	6.7837(1)	332.71(1)	89.0(2)
		UO_2	5.4604(2)			162.81(2)	11.0(2)
**53**	749	UO_2	5.4597(3)			162.74(2)	8.6(2)
		U_3O_8	4.13743(8)	11.8604(2)	6.7834(1)	332.87(1)	91.4(2)
**54**	724	UO_2	5.4585(4)			162.64(4)	5.8(1)
		U_3O_8	4.13873(9)	11.8636(2)	6.7835(1)	333.1(1)	94.2(1)
**55**	699	U_3O_8	4.13934(1)	11.8656(2)	6.7823(1)	333.12(1)	96.2(1)
		UO_2	5.4559(6)			162.40(6)	3.8(1)
**56**	674	UO_2	5.4528(8)			162.13(7)	2.44(1)
		U_3O_8	4.13965(1)	11.8661(3)	6.7803(1)	333.06(1)	97.56(1)
**57**	649	UO_2	5.453(1)			162.16(1)	1.78(9)
		U_3O_8	4.14004(1)	11.8658(3)	6.7787(2)	333.00(1)	98.22(9)
**58**	624	U_3O_8	4.14021(1)	11.8653(3)	6.7770(1)	332.92(1)	98.45(8)
		UO_2	5.450(1)			161.9(1)	1.55(8)
**59**	599	UO_2	5.448(1)			161.7(1)	1.60(9)
		U_3O_8	4.14071(1)	11.8641(3)	6.7756(2)	332.86(1)	98.40(9)
**60**	574	U_3O_8	4.14156(9)	11.8632(3)	6.7751(2)	332.87(1)	98.23(1)
		UO_2	5.447(1)			162(1)	1.77(1)
**61**	549	U_3O_8	4.14218(9)	11.8620(3)	6.7731(2)	332.79(1)	98.2(1)
		UO_2	5.445(1)			161.4(1)	1.8(1)
**62**	524	U_3O_8	4.1428(9)	11.8625(3)	6.7711(2)	332.76(1)	98.40(1)
		UO_2	5.446(1)			161.6(1)	1.60(1)
**63**	499	U_3O_8	4.14316(9)	11.8626(3)	6.7685(2)	332.66(1)	98.34(1)
		UO_2	5.444(1)			161.3(1)	1.66(1)
**64**	474	U_3O_8	4.14369(9)	11.8640(3)	6.7661(2)	332.62(1)	98.3(1)
		UO_2	5.440(1)			161.0(1)	1.7(1)
**65**	449	U_3O_8	4.14387(8)	11.8661(3)	6.7633(2)	332.56(1)	98.3(1)
		UO_2	5.439(1)			160.9(1)	1.7(1)
**66**	424	U_3O_8	4.14438(9)	11.8687(3)	6.7602(2)	332.52(1)	98.2(1)
		UO_2	5.436(2)			160.6(1)	1.8(1)
**67**	399	U_3O_8	4.14462(8)	11.8716(3)	6.7570(2)	332.47(1)	98.3(1)
		UO_2	5.434(1)			160.4(1)	1.7(1)
**68**	374	U_3O_8	4.14511(8)	11.8761(3)	6.7538(2)	332.47(1)	98.2(1)
		UO_2	5.435(2)			160.6(1)	1.8(1)
**69**	349	U_3O_8	4.14540(8)	11.8807(3)	6.7501(2)	332.45(1)	98.3(1)
		UO_2	5.435(2)			160.6(1)	1.7(1)
**70**	324	U_3O_8	4.14572(8)	11.8853(3)	6.7464(2)	332.42(1)	98.2(1)
		UO_2	5.433(2)			160.3(2)	1.8(1)
**71**	299	UO_2	5.43(2)			160.4(2)	1.7(1)
		U_3O_8	4.14619(8)	11.8906(2)	6.7422(1)	332.39(1)	98.3(1)

2. U_3O_8 in a helium atmosphere

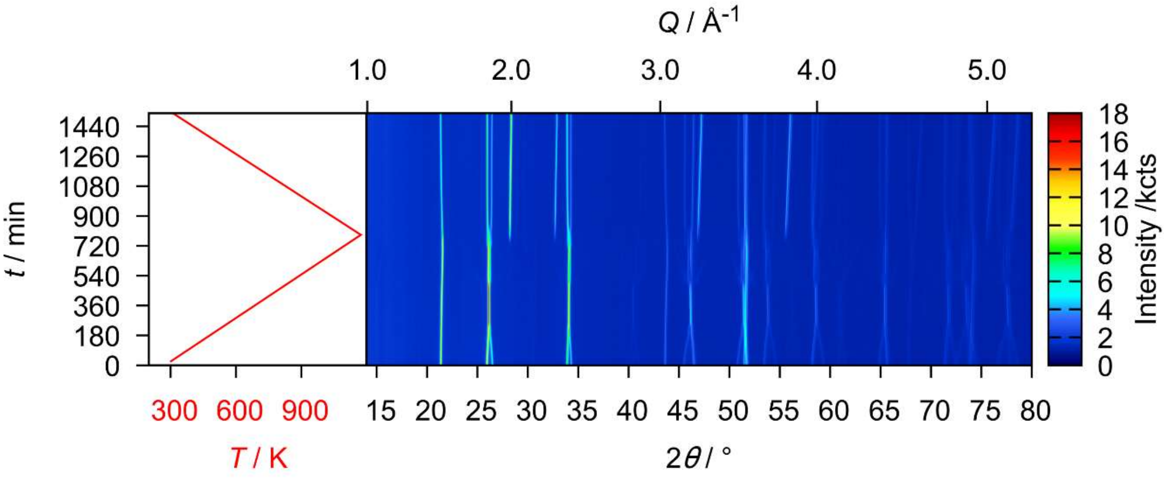

Figure S2: False-color plot of the *in situ* X-ray powder diffraction (Cu-$K_{\bar{\alpha}}$ radiation, BB geometry) data of U_3O_8 between room temperature and 1073 K in a static atmosphere of helium.

Table S2: Refined lattice parameters and phase fractions of all phases appearing in the *in situ* X-ray diffraction experiment of U_3O_8 in an atmosphere of helium.

ID	*T* / K	phase	*a* / Å	*b* / Å	*c* / Å	*V* / Å^3	*ω* / wt.-%
**0**	298	U_3O_8	4.14848(5)	11.9606(2)	6.7210(1)	333.485(8)	100
**1**	322	U_3O_8	4.14848(5)	11.9531(2)	6.7256(1)	333.504(8)	100
**2**	347	U_3O_8	4.14846(5)	11.9442(2)	6.7311(1)	333.525(8)	100
**3**	372	U_3O_8	4.14843(5)	11.9341(2)	6.7367(1)	333.519(8)	100
**4**	397	U_3O_8	4.14840(5)	11.9233(2)	6.7428(1)	333.516(8)	100
**5**	422	U_3O_8	4.14831(5)	11.9119(2)	6.7492(1)	333.507(8)	100
**6**	447	U_3O_8	4.14818(5)	11.8995(2)	6.7561(1)	333.488(8)	100
**7**	472	U_3O_8	4.14786(5)	11.8849(2)	6.7643(1)	333.458(8)	100
**8**	497	U_3O_8	4.14758(5)	11.8636(2)	6.7756(1)	333.393(8)	100
**9**	522	U_3O_8	4.14721(5)	11.8490(2)	6.7844(1)	333.389(8)	100
**10**	523	U_3O_8	4.14667(5)	11.8342(2)	6.7931(1)	333.358(9)	100
**11**	547	U_3O_8	4.14592(4)	11.8064(6)	6.8093(4)	333.30(2)	100
**12**	572	U_3O_8	4.14535(4)	11.8040(4)	6.8118(3)	333.31(2)	100
**13**	597	U_3O_8	4.14494(4)	11.8053(4)	6.8133(3)	333.39(2)	100
**14**	622	U_3O_8	4.14458(4)	11.8070(4)	6.8142(3)	333.46(2)	100
**15**	647	U_3O_8	4.14420(4)	11.8087(4)	6.8153(3)	333.53(2)	100
**16**	672	U_3O_8	4.14391(4)	11.8107(5)	6.8166(4)	333.62(2)	100
**17**	697	U_3O_8	4.14359(4)	11.8129(4)	6.8176(4)	333.71(2)	100
**18**	722	U_3O_8	4.14333(4)	11.8147(4)	6.8191(3)	333.81(2)	100
**19**	747	U_3O_8	4.14300(4)	11.8176(3)	6.8197(2)	333.90(2)	100
**20**	772	U_3O_8	4.14271(4)	11.8189(4)	6.8216(3)	334.00(2)	100
**21**	797	U_3O_8	4.14243(4)	11.8212(3)	6.8229(3)	334.11(2)	100
**22**	822	U_3O_8	4.14182(6)	11.8288(5)	6.8210(4)	334.18(2)	100
**23**	847	U_3O_8	4.14140(8)	11.8652(3)	6.8017(2)	334.22(2)	100
**24**	872	U_3O_8	4.14025(8)	11.8794(2)	6.7974(2)	334.32(1)	100
**25**	897	U_3O_8	4.13970(7)	11.8815(2)	6.7991(1)	334.42(1)	100
**26**	922	U_3O_8	4.13936(6)	11.8807(2)	6.8009(1)	334.456(9)	100
**27**	947	U_3O_8	4.13906(6)	11.8791(2)	6.8027(1)	334.478(9)	100
**28**	972	U_3O_8	4.13895(6)	11.8785(2)	6.80462(1)	334.544(8)	100
**29**	997	U_3O_8	4.13849(6)	11.8771(1)	6.80657(1)	334.565(8)	100
**30**	1022	U_3O_8	4.13806(6)	11.8758(1)	6.80785(9)	334.557(8)	100
**31**	1047	U_3O_8	4.13782(5)	11.8757(1)	6.80862(8)	334.572(7)	100
**32**	1072	U_3O_8	4.13785(5)	11.8780(1)	6.80856(8)	334.636(7)	100
**33**	1097	U_3O_8	4.13786(7)	11.8803(2)	6.81018(1)	334.781(9)	67.5(6)
		U_3O_{8-x}	4.1440(1)	11.9588(3)	6.7744(2)	335.73(2)	32.5(6)
**34**	1122	U_3O_8	4.13786(8)	11.8821(2)	6.8116(1)	334.90(1)	53.3(6)
		U_3O_{8-x}	4.1435(1)	11.9594(3)	6.7769(2)	335.83(1)	42.3(6)
		UO_2	5.49142(2)			165.60(2)	4.4(1)
**35**	1147	U_3O_8	4.13785(8)	11.8843(2)	6.8136(1)	335.06(1)	49.0(6)
		U_3O_{8-x}	4.1434(1)	11.9602(3)	6.7789(2)	335.94(1)	40.5(6)
		UO_2	5.49431(9)			165.859(8)	10.4(2)
**36**	1172	U_3O_8	4.13767(8)	11.8816(2)	6.8117(1)	334.88(1)	44.4(6)
		U_3O_{8-x}	4.1433(1)	11.9592(3)	6.7765(2)	335.78(2)	38.0(6)
		UO_2	5.49186(8)			165.638(6)	17.6(3)
**37**	1149	U_3O_8	4.13755(9)	11.8798(2)	6.8095(1)	334.71(1)	39.0(6)
		U_3O_{8-x}	4.14330(1)	11.9582(3)	6.7741(2)	335.63(1)	37.4(6)
		UO_2	5.48965(6)			165.438(5)	23.5(4)
**38**	1124	U_3O_8	4.1380(2)	11.8813(6)	6.8040(4)	334.52(3)	21.2(5)
		U_3O_{8-x}	4.14365(7)	11.9570(2)	6.7718(1)	335.51(1)	51.3(6)
		UO_2	5.48766(5)			165.257(5)	27.6(4)
**39**	1099	U_3O_8	4.13819(6)	11.897(2)	6.793(1)	334.4(1)	9.2(6)
		U_3O_{8-x}	4.14365(6)	11.9559(2)	6.7700(1)	335.39(9)	59.6(7)
		UO_2	5.48544(5)			165.057(4)	31.2(5)
**40**	1074	U_3O_{8-x}	4.14393(5)	11.9545(2)	6.76799(9)	335.278(8)	65.5(5)
		UO_2	5.48352(4)			164.884(4)	34.5(5)
**41**	1049	U_3O_{8-x}	4.14428(6)	11.9546(2)	6.76588(9)	335.204(8)	64.1(5)

		UO_2	5.48166(4)			164.716(4)	35.9(5)
**42**	1024	U_3O_{8-x}	4.14465(6)	11.9555(2)	6.76325(9)	335.128(8)	62.8(5)
		UO_2	5.47974(4)			164.543(4)	37.2(5)
**43**	999	U_3O_{8-x}	4.14516(6)	11.9572(2)	6.76047(9)	335.080(8)	61.8(5)
		UO_2	5.47799(4)			164.385(4)	38.2(5)
**44**	974	U_3O_{8-x}	4.14560(6)	11.9581(2)	6.75785(1)	335.012(8)	61.7(5)
		UO_2	5.47615(4)			164.220(4)	38.3(5)
**45**	949	U_3O_{8-x}	4.14604(6)	11.9583(2)	6.75579(1)	334.949(8)	61.3(5)
		UO_2	5.47451(4)			164.073(4)	38.7(5)
**46**	924	U_3O_{8-x}	4.14610(6)	11.9573(2)	6.75386(9)	334.830(8)	61.1(5)
		UO_2	5.47267(4)			163.907(4)	38.9(5)
**47**	899	U_3O_{8-x}	4.14645(6)	11.9563(2)	6.75234(9)	334.755(8)	61.0(5)
		UO_2	5.47115(4)			163.771(4)	39.0(5)
**48**	874	U_3O_{8-x}	4.14668(6)	11.9548(2)	6.75091(9)	334.661(8)	61.0(5)
		UO_2	5.46952(4)			163.625(4)	39.0(5)
**49**	849	U_3O_{8-x}	4.14675(6)	11.9531(2)	6.74937(9)	334.543(8)	60.8(5)
		UO_2	5.46779(4)			163.469(4)	39.2(5)
**50**	824	U_3O_{8-x}	4.14694(6)	11.9514(2)	6.74836(9)	334.460(8)	60.8(5)
		UO_2	5.46620(4)			163.326(4)	39.2(5)
**51**	799	U_3O_{8-x}	4.14718(6)	11.949287(163	6.74707(9)	334.356(8)	60.9(5)
		UO_2	5.46459(4)			163.182(4)	39.1(5)
**52**	UO_2	U_3O_{8-x}	4.14705(6)	11.9474(2)	6.74555(9)	334.217(8)	60.8(5)
		UO_2	5.46273(4)			163.016(4)	39.2(5)
**53**	749	U_3O_{8-x}	4.14722(6)	11.9452(2)	6.74451(9)	334.121(8)	60.8(5)
		UO_2	5.46109(4)			162.869(4)	39.2(5)
**54**	724	U_3O_{8-x}	4.14724(6)	11.9436(2)	6.74317(9)	334.009(8)	60.7(5)
		UO_2	5.45937(4)			162.715(4)	39.3(5)
**55**	699	U_3O_{8-x}	4.14745(6)	11.9422(2)	6.74206(9)	333.931(8)	60.7(5)
		UO_2	5.45784(4)			162.578(4)	39.3(5)
**56**	674	U_3O_{8-x}	4.14738(6)	11.9403(2)	6.74091(9)	333.818(8)	60.6(5)
		UO_2	5.45608(4)			162.421(4)	39.4(5)
**57**	649	U_3O_{8-x}	4.14759(6)	11.9389(2)	6.73955(9)	333.728(8)	60.6(5)
		UO_2	5.45449(4)			162.279(4)	39.4(5)
**58**	624	U_3O_{8-x}	4.14780(6)	11.9374(2)	6.73873(9)	333.662(8)	60.6(5)
		UO_2	5.4530(4)			162.144(4)	39.4(5)
**59**	599	U_3O_{8-x}	4.14775(6)	11.9359(2)	6.73715(9)	333.538(8)	60.5(5)
		UO_2	5.45125(4)			161.990(4)	39.5(5)
**60**	574	U_3O_{8-x}	4.14797(6)	11.9347(2)	6.73601(1)	333.465(8)	60.5(5)
		UO_2	5.44984(4)			161.864(4)	39.5(5)
**61**	549	U_3O_{8-x}	4.14799(6)	11.9332(2)	6.73483(9)	333.366(8)	60.3(5)
		UO_2	5.44825(4)			161.723(4)	39.7(5)
**62**	524	U_3O_{8-x}	4.14816(6)	11.9327(2)	6.73369(1)	333.310(8)	60.2(5)
		UO_2	5.44686(4)			161.599(4)	39.8(5)
**63**	499	U_3O_{8-x}	4.14823(6)	11.9311(2)	6.73245(1)	333.209(8)	60.4(5)
		UO_2	5.44537(4)			161.467(4)	39.6(5)
**64**	474	U_3O_{8-x}	4.14837(6)	11.9304(2)	6.73120(9)	333.140(8)	60.3(5)
		UO_2	5.44396(4)			161.341(4)	39.7(5)
**65**	449	U_3O_{8-x}	4.14834(6)	11.9291(2)	6.72979(1)	333.030(8)	60.3(5)
		UO_2	5.44237(4)			161.200(4)	39.7(5)
**66**	424	U_3O_{8-x}	4.14844(6)	11.9283(2)	6.72863(1)	332.957(8)	60.3(5)
		UO_2	5.44109(4)			161.086(4)	39.7(5)
**67**	399	U_3O_{8-x}	4.14857(6)	11.9274(2)	6.72731(1)	332.879(8)	60.4(5)
		UO_2	5.43974(4)			160.966(4)	39.6(5)
**68**	374	U_3O_{8-x}	4.14875(6)	11.9270(2)	6.72612(1)	332.823(8)	60.3(5)
		UO_2	5.43864(4)			160.868(4)	39.7(5)
**69**	349	U_3O_{8-x}	4.14874(6)	11.9261(2)	6.72467(1)	332.725(8)	60.2(5)
		UO_2	5.43784(5)			160.797(4)	39.8(5)
**70**	324	U_3O_{8-x}	4.14896(6)	11.9256(2)	6.72363(1)	332.677(8)	60.1(5)
		UO_2	5.43786(5)			160.799(4)	39.9(5)

3. U_3O_8 reduction by hydrogen gas

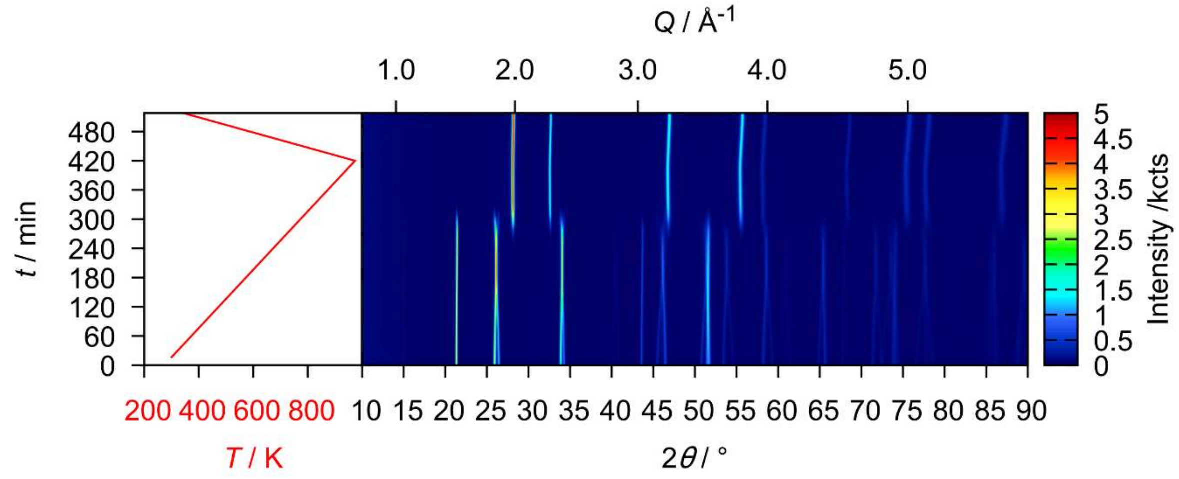

Figure S3: False-color plot of part of the *in situ* X-ray powder diffraction (Cu-$K_{\bar{\alpha}}$ radiation, PB geometry) data of U_3O_8 between room temperature and 973 K in a flow of 10 sccm of hydrogen under a pressure of 1.0 bar.

Table S3: Refined lattice parameters and phase fractions of all phases appearing in the *in situ* X-ray diffraction experiment of the reduction of U_3O_8 by hydrogen gas.

ID	*T* / K	phase	*a* / Å	*b* / Å	*c* / Å	*V* / Å³	*ω* / wt.-%
**0**	297	U_3O_8	4.14568(7)	11.9475(2)	6.7190(1)	332.80(1)	100
**1**	322	U_3O_8	4.14569(7)	11.9400(2)	6.7240(1)	332.83(1)	100
**2**	347	U_3O_8	4.14564(8)	11.9312(2)	6.7291(1)	332.84(1)	100
**3**	372	U_3O_8	4.14573(8)	11.9216(2)	6.7350(1)	332.87(1)	100
**4**	397	U_3O_8	4.14543(8)	11.9110(2)	6.7405(1)	332.82(1)	100
**5**	422	U_3O_8	4.14532(8)	11.9000(2)	6.7469(1)	332.82(1)	100
**6**	447	U_3O_8	4.14497(8)	11.8873(2)	6.7535(1)	332.76(1)	100
**7**	472	U_3O_8	4.14479(8)	11.8743(2)	6.7607(1)	332.74(1)	100
**8**	497	U_3O_8	4.14452(8)	11.8552(2)	6.7712(1)	332.70(1)	100
**9**	522	U_3O_8	4.14401(8)	11.8426(2)	6.7787(1)	332.67(1)	100
**10**	547	U_3O_8	4.14345(8)	11.8297(3)	6.7867(2)	332.65(1)	100
**11**	572	U_3O_8	4.14276(7)	11.810(1)	6.7986(6)	332.63(4)	100
**12**	597	U_3O_8	4.14213(7)	11.803(1)	6.8035(8)	332.62(6)	100
**13**	622	U_3O_8	4.14162(7)	11.7983(6)	6.8082(4)	332.67(3)	100
**14**	647	U_3O_8	4.14117(7)	11.8006(5)	6.8092(4)	332.75(2)	100
**15**	672	U_3O_8	4.14066(7)	11.8019(4)	6.8103(4)	332.80(2)	100
**16**	697	U_3O_8	4.14025(8)	11.8055(4)	6.8108(4)	332.90(2)	98.75(6)
		UO_2	5.4832(5)			164.85(5)	1.25(6)
**17**	722	U_3O_8	4.13973(9)	11.8119(5)	6.8090(4)	332.94(2)	95.8(2)
		UO_2	5.4842(2)			164.94(2)	4.2(2)
**18**	747	U_3O_8	4.1385(1)	11.8539(1)	6.7852(6)	332.87(4)	80.7(5)
		U_3O_{8-x}	4.1460(4)	11.77(2)	6.8207(7)	332.8(5)	3.9(5)
		UO_2	5.4850(2)			165.02(1)	15.4(2)
**19**	772	U_3O_8	4.1466(3)	11.9655(9)	6.7364(5)	334.23(4)	27.8(4)
		U_3O_{8-x}	4.1427(2)	11.859(1)	6.7814(8)	333.17(6)	26.8(4)
		UO_2	5.4878(1)			165.27(1)	45.5(4)
**20**	797	U_3O_8	4.1493(2)	11.9704(7)	6.7399(3)	334.76(3)	20.0(8)
		UO_2	5.4903(1)			165.50(1)	80.0(8)
**21**	822	U_3O_8	4.1507(4)	11.982(2)	6.740(1)	335.20(8)	4.4(3)
		UO_2	5.4928(1)			165.72(1)	95.6(3)
**22**	847	UO_2	5.4947(1)			165.89(1)	100
**23**	872	UO_2	5.4964(1)			166.05(1)	100
**24**	897	UO_2	5.4979(1)			166.188(9)	100
**25**	922	UO_2	5.4996(1)			166.342(9)	100
**26**	947	UO_2	5.5010(1)			166.466(9)	100

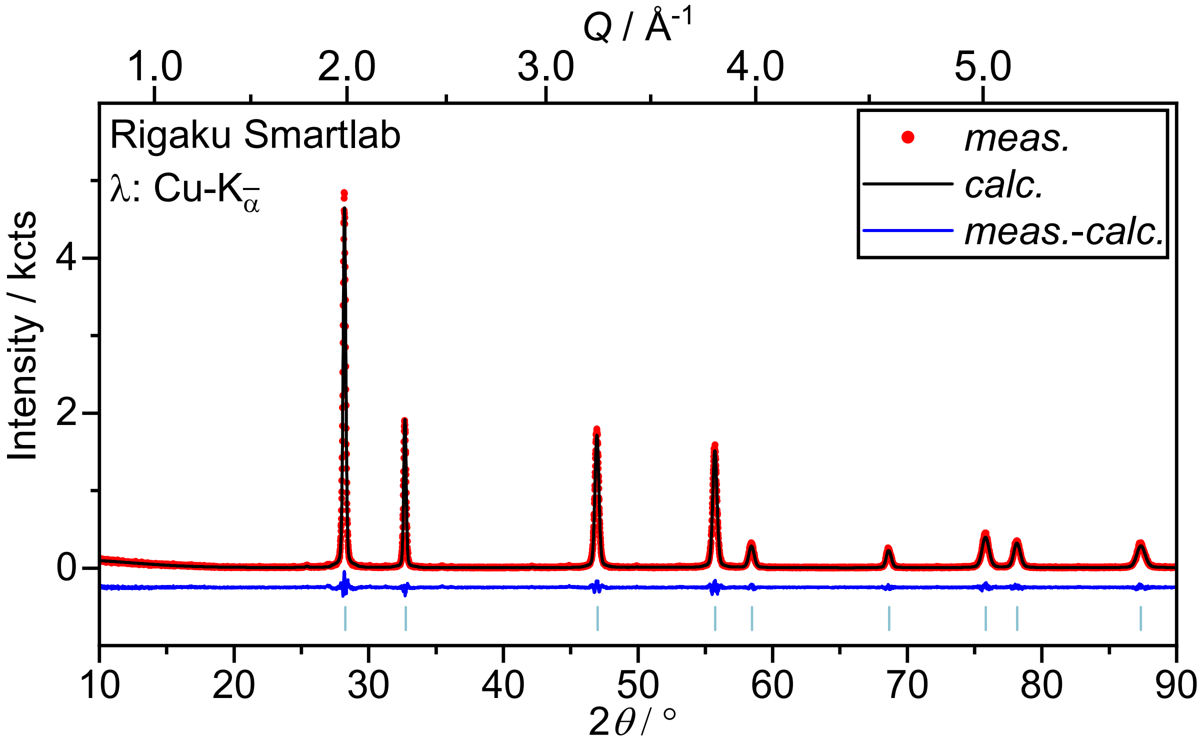

Figure S4: Rietveld refinement of the crystal structure of UO_2 based on X-ray diffraction data collected after reducing U_3O_8 by hydrogen gas (R_{wp} = 14.41 %, *GoF* = 1.16). Bragg markers denote from top to bottom: UO_2 ($Fm\overline{3}m$, R_{Bragg} = 1.315 %, *a* = 5.46518(9) Å, 100 wt.-%).

4. Partial reduction of U_3O_8 by a mixture of hydrogen and argon gas

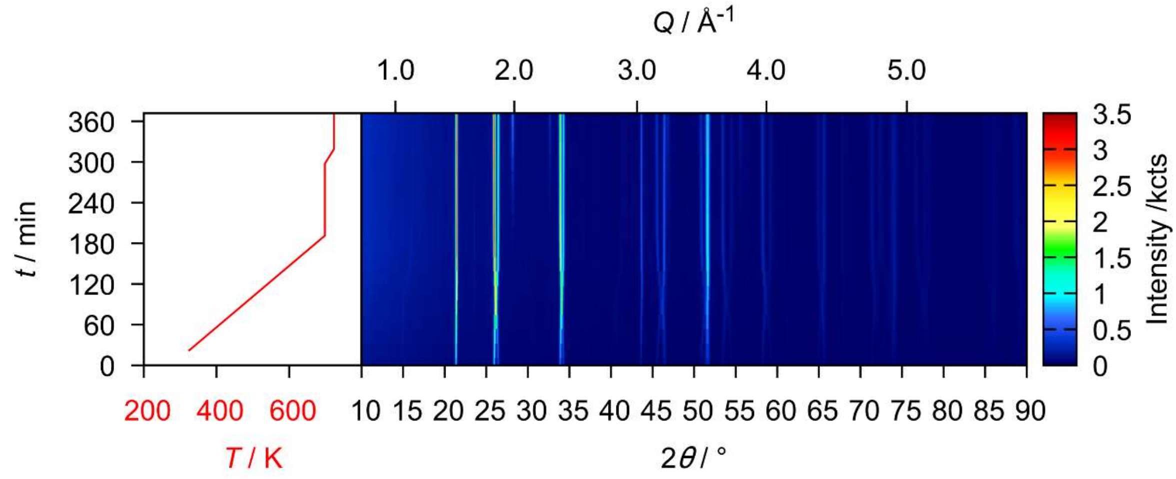

Figure S5: False-color plot of the *in situ* X-ray powder diffraction (Cu-$K_{\bar{\alpha}}$ radiation, PB geometry) data of U_3O_8 between room temperature and 723 K in a flow of 10 sccm of an argon hydrogen mixture (5 vol.-% hydrogen) under a pressure of 1.3 bar.

Table S4: Refined lattice parameters and phase fractions of all phases appearing in the *in situ* X-ray diffraction experiment of the partial reduction of U_3O_8 by a mixture of argon and hydrogen gas.

ID	*T* / K	phase	*a* / Å	*b* / Å	*c* / Å	*V* / Å^3	*ω* / wt.-%
**0**	322	U_3O_8	4.1463(2)	11.9431(5)	6.7263(3)	333.09(2)	100
**1**	372	U_3O_8	4.1462(1)	11.9240(4)	6.7365(3)	333.05(2)	100
**2**	422	U_3O_8	4.1460(1)	11.9009(4)	6.7503(2)	333.06(2)	100
**3**	472	U_3O_8	4.1455(1)	11.8693(4)	6.7668(2)	332.96(2)	100
**4**	522	U_3O_8	4.1439(1)	11.8333(4)	6.7868(3)	332.80(2)	100
**5**	572	U_3O_8	4.1414(1)	11.8455(4)	6.7807(3)	332.64(2)	100
**6**	622	U_3O_8	4.1422(1)	11.8806(3)	6.7645(2)	332.89(2)	100
**7**	672	U_3O_8	4.14557(1)	11.9239(3)	6.7504(2)	333.68(1)	100
**8**	697	U_3O_8	4.14668(9)	11.9404(3)	6.7473(2)	334.08(1)	100
**9**	698	U_3O_8	4.14657(9)	11.9478(3)	6.7447(2)	334.15(1)	100
**10**	698	U_3O_8	4.14655(9)	11.9509(3)	6.7433(2)	334.17(1)	98.59(7)
		UO_2	5.4841(8)			164.94(7)	1.41(7)
**11**	698	U_3O_8	4.14691(9)	11.9536(3)	6.7425(2)	334.23(1)	97.94(7)
		UO_2	5.4842(5)			164.94(4)	2.06(7)
**12**	698	U_3O_8	4.14701(9)	11.9553(3)	6.7420(2)	334.26(1)	96.94(7)
		UO_2	5.4849(4)			165.01(4)	3.06(7)
**13**	698	U_3O_8	4.14710(9)	11.9560(3)	6.7413(2)	334.25(1)	96.42(7)
		UO_2	5.4835(3)			164.88(3)	3.58(7)
**14**	722	U_3O_8	4.14722(9)	11.9604(3)	6.7408(2)	334.36(1)	94.73(8)
		UO_2	5.4855(2)			165.06(2)	5.27(8)
**15**	723	U_3O_8	4.14729(9)	11.9620(3)	6.7396(2)	334.35(1)	93.50(8)
		UO_2	5.4855(2)			165.06(2)	6.50(8)
**16**	723	U_3O_8	4.14758(9)	11.9641(3)	6.7392(2)	334.41(1)	92.15(8)
		UO_2	5.4855(2)			165.06(2)	7.85(8)
**17**	723	U_3O_8	4.14775(9)	11.9647(3)	6.7386(2)	334.41(1)	90.7(1)
		UO_2	5.4858(2)			165.09(2)	9.3(1)

5. Reduction of U_3O_8 by PVDF

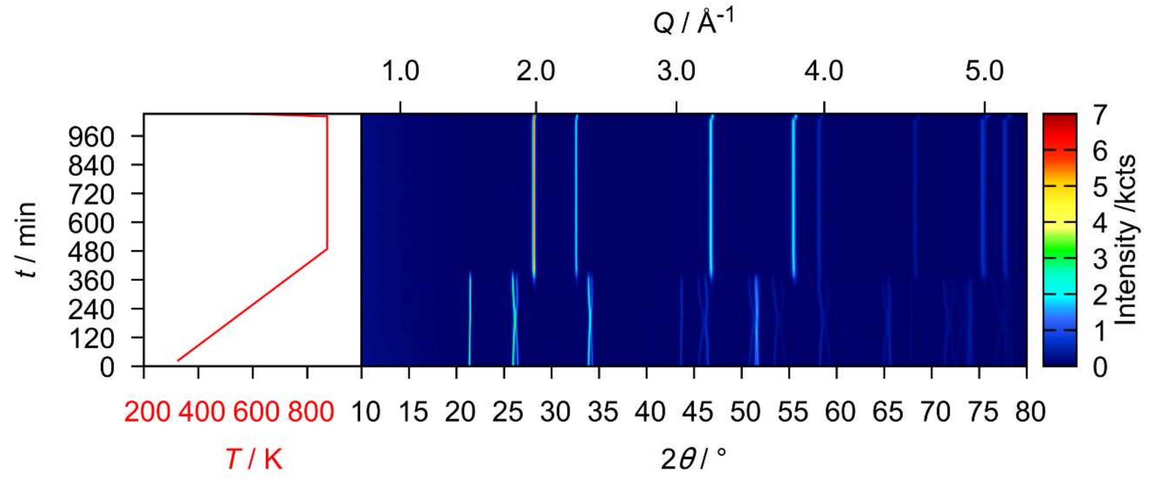

Figure S6: False-color plot of part of the *in situ* X-ray powder diffraction (Cu-K$_{\bar{\alpha}}$ radiation, PB geometry) data of a mixture of U_3O_8 and PVDF between room temperature and 873 K in a dynamic vacuum.

Table S5: Refined lattice parameters and phase fractions of all phases appearing in the *in situ* X-ray diffraction experiment of the reduction of U_3O_8 by PVDF.

ID	*T* / K	phase	*a* / Å	*b* / Å	*c* / Å	*V* / Å^3	*ω* / wt.-%
**0**	322	U_3O_8	4.14734(7)	11.9489(2)	6.7247(1)	333.25(1)	100
**1**	347	U_3O_8	4.14727(7)	11.9410(2)	6.7293(1)	333.25(1)	100
**2**	372	U_3O_8	4.14726(7)	11.9310(2)	6.7349(1)	333.25(1)	100
**3**	397	U_3O_8	4.14728(7)	11.9209(2)	6.7409(1)	333.27(1)	100
**4**	422	U_3O_8	4.14719(7)	11.9096(2)	6.7474(1)	333.26(1)	100
**5**	447	U_3O_8	4.14705(7)	11.8976(2)	6.7544(1)	333.26(1)	100
**6**	472	U_3O_8	4.14688(8)	11.8844(2)	6.7622(1)	333.26(1)	100
**7**	497	U_3O_8	4.14613(8)	11.8647(2)	6.7725(1)	333.15(1)	100
**8**	522	U_3O_8	4.14449(9)	11.8361(3)	6.7873(2)	332.95(2)	100
**9**	547	U_3O_8	4.14293(1)	11.8403(3)	6.7861(2)	332.88(2)	100
**10**	572	U_3O_8	4.14174(1)	11.8542(3)	6.7784(2)	332.80(1)	100
**11**	597	U_3O_8	4.14205(9)	11.8696(3)	6.7717(2)	332.93(1)	100
**12**	622	U_3O_8	4.14607(9)	11.9124(3)	6.7567(2)	333.71(1)	100
**13**	647	U_3O_8	4.14693(8)	11.9323(3)	6.7531(2)	334.16(1)	100
**14**	672	U_3O_8	4.14768(9)	11.9471(3)	6.7546(2)	334.71(1)	100
**15**	697	U_3O_8	4.14715(9)	11.9626(3)	6.7591(2)	335.33(1)	96.2(3)
		UO_2	5.4843(3)			164.96(3)	3.8(3)
**16**	722	U_3O_8	4.14497(9)	11.9733(3)	6.7685(2)	335.92(1)	90.6(5)
		UO_2	5.48715(2)			165.21(2)	9.4(5)
**17**	747	U_3O_8	4.14389(1)	11.9789(3)	6.7701(2)	336.06(2)	74(4)
		UO_2	5.4891(1)			165.38(1)	26(4)
**18**	772	U_3O_8	4.1467(1)	11.989(5)	6.7560(4)	335.86(2)	30(5)
		UO_2	5.4919(1)			165.64(1)	70(5)
**19**	797	UO_2	5.4934(1)			165.78(1)	100
**20**	822	UO_2	5.4949(1)			165.917(9)	100
**21**	847	UO_2	5.4968(1)			166.085(9)	100
**22**	872	UO_2	5.49825(1)			166.216(9)	100
**23**	873	UO_2	5.49833(1)			166.223(9)	100
**24**	873	UO_2	5.49829(1)			166.220(9)	100
**25**	873	UO_2	5.49831(1)			166.222(9)	100
**26**	873	UO_2	5.49842(1)			166.232(9)	100
**27**	873	UO_2	5.49838(1)			166.228(9)	100
**28**	873	UO_2	5.49828(1)			166.219(9)	100
**29**	873	UO_2	5.49828(1)			166.219(9)	100
**30**	873	UO_2	5.49846(1)			166.235(9)	100
**31**	873	UO_2	5.49837(1)			166.227(9)	100
**32**	873	UO_2	5.49849(1)			166.238(9)	100
**33**	873	UO_2	5.49856(1)			166.244(9)	100
**34**	873	UO_2	5.49840(9)			166.230(9)	100
**35**	873	UO_2	5.49860(9)			166.248(9)	100
**36**	873	UO_2	5.49845(1)			166.234(9)	100
**37**	873	UO_2	5.49848(9)			166.237(9)	100
**38**	873	UO_2	5.49849(9)			166.238(9)	100
**39**	873	UO_2	5.49845(9)			166.235(9)	100
**40**	873	UO_2	5.49848(9)			166.237(8)	100
**41**	873	UO_2	5.49851(9)			166.240(9)	100
**42**	873	UO_2	5.49846(9)			166.235(8)	100
**43**	873	UO_2	5.49849(9)			166.238(8)	100
**44**	873	UO_2	5.49854(9)			166.242(8)	100
**45**	873	UO_2	5.49854(9)			166.242(8)	100
**46**	873	UO_2	5.49854(9)			166.243(8)	100
**47**	873	UO_2	5.49857(9)			166.245(8)	100
**48**	873	UO_2	5.49850(9)			166.239(8)	100
**49**	299	UO_2	5.46561(9)			163.274(8)	100

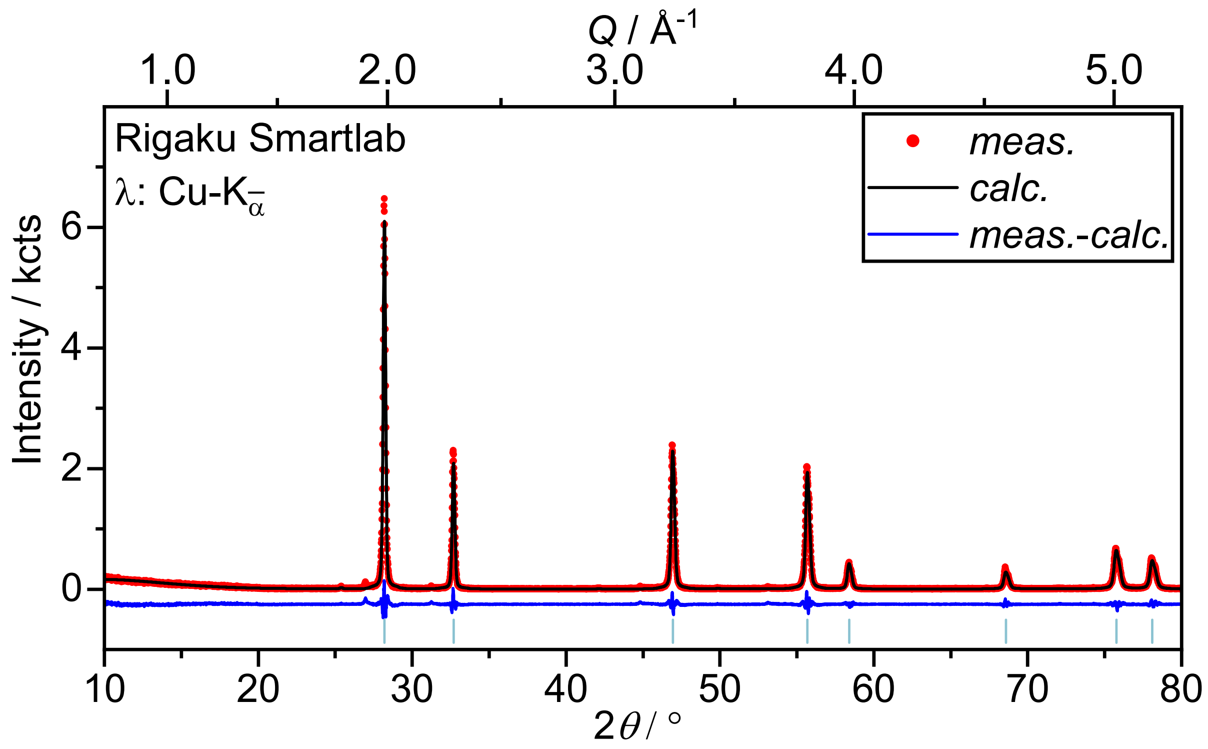

Figure S7: Rietveld refinement of the crystal structure of UO_2 based on X-ray diffraction data collected after reducing U_3O_8 by PVDF (R_{wp} = 16.51 %, *GoF* = 1.47). Bragg markers denote from top to bottom: UO_2 ($Fm\overline{3}m$, R_{Bragg} = 1.188 %, *a* = 5.46561(9) Å, 100 wt.-%).

6. U_3O_8 in a dynamic vacuum

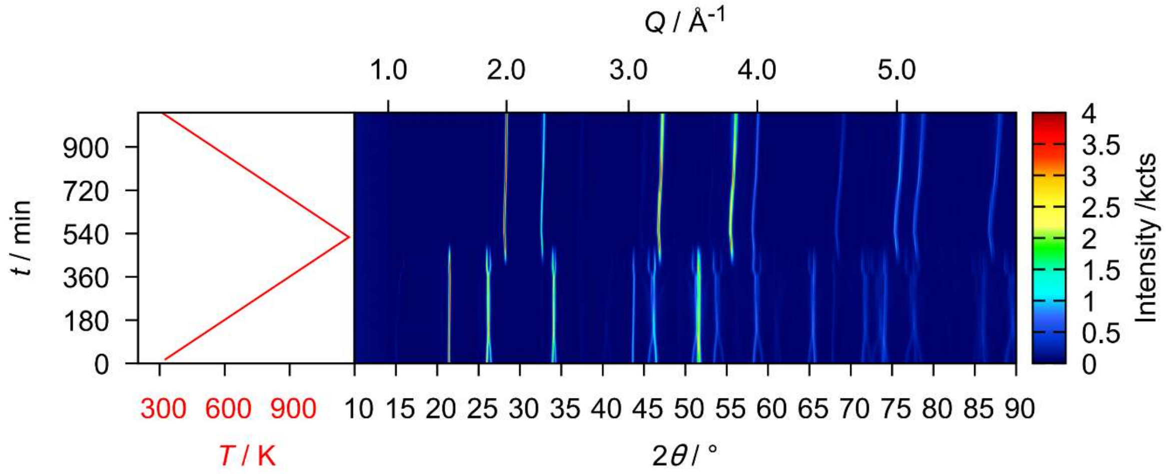

Figure S8: False-color plot of the *in situ* X-ray powder diffraction (Cu-K$_{\bar{\alpha}}$ radiation, PB geometry) data of U_3O_8 in a dynamic vacuum between room temperature and 1173 K.

Table S6: Refined lattice parameters and phase fractions of all phases appearing in the *in situ* X-ray diffraction experiment of U_3O_8 in a dynamic vacuum.

ID	*T* / K	phase	*a* / Å	*b* / Å	*c* / Å	*V* / Å^3	*ω* / wt.-%
**0**	325	U_3O_8	4.14866(8)	11.9520(2)	6.7265(1)	333.53(1)	100
**1**	348	U_3O_8	4.14883(8)	11.9424(2)	6.7325(1)	333.58(1)	100
**2**	373	U_3O_8	4.14881(8)	11.9314(2)	6.7387(1)	333.57(1)	100
**3**	398	U_3O_8	4.14870(8)	11.9192(2)	6.7457(1)	333.57(1)	100
**4**	423	U_3O_8	4.14855(8)	11.9064(2)	6.7528(1)	333.55(1)	100
**5**	448	U_3O_8	4.14832(9)	11.8910(2)	6.7612(1)	333.51(1)	100
**6**	473	U_3O_8	4.1481(1)	11.8668(3)	6.7744(2)	333.46(1)	100
**7**	498	U_3O_8	4.1475(1)	11.8519(3)	6.7837(2)	333.46(1)	100
**8**	523	U_3O_8	4.1469(1)	11.8400(3)	6.7916(2)	333.46(2)	100
**9**	548	U_3O_{8-x}	4.1456(6)	11.854(1)	6.7788(7)	333.11(7)	25(2)
		U_3O_8	4.1462(1)	11.8050(4)	6.8142(2)	333.53(2)	75(2)
**10**	573	U_3O_{8-x}	4.1435(5)	11.870(1)	6.7737(7)	333.15(6)	35(2)
		U_3O_8	4.1455(1)	11.8055(4)	6.8142(2)	333.49(2)	65(2)
**11**	598	U_3O_{8-x}	4.1432(5)	11.873(1)	6.7736(6)	333.20(6)	37(2)
		U_3O_8	4.1449(1)	11.8077(4)	6.8153(2)	333.55(2)	63(2)
**12**	623	U_3O_{8-x}	4.1425(5)	11.872(1)	6.7750(6)	333.20(6)	40(2)
		U_3O_8	4.1444(1)	11.8096(4)	6.8162(2)	333.61(2)	60(2)
**13**	648	U_3O_{8-x}	4.1429(5)	11.873(1)	6.7757(6)	333.29(6)	42(2)
		U_3O_8	4.1438(1)	11.8119(4)	6.8176(2)	333.70(2)	58(2)
**14**	673	U_3O_{8-x}	4.1423(5)	11.8747(9)	6.7770(5)	333.36(5)	43(2)
		U_3O_8	4.1433(1)	11.8145(5)	6.8189(2)	333.80(2)	57(2)
**15**	698	U_3O_8	4.1427(1)	11.8190(6)	6.8204(3)	333.95(2)	54(2)
		U_3O_{8-x}	4.1428(5)	11.8751(9)	6.7792(5)	333.51(5)	46(2)
**16**	723	U_3O_{8-x}	4.1415(3)	11.8713(6)	6.7838(3)	333.53(3)	69(1)
		U_3O_8	4.1421(2)	11.8186(4)	6.8221(3)	333.97(2)	31(1)
**17**	748	U_3O_{8-x}	4.1414(3)	11.8732(5)	6.7848(3)	333.62(3)	74(1)
		U_3O_8	4.1411(2)	11.8228(6)	6.8238(4)	334.09(3)	26(1)
**18**	773	U_3O_{8-x}	4.1409(2)	11.8759(5)	6.7861(3)	333.71(3)	73(1)
		U_3O_8	4.1396(3)	11.8366(6)	6.8300(4)	334.67(3)	27(1)
**19**	798	U_3O_{8-x}	4.1403(2)	11.8754(5)	6.7883(2)	333.77(2)	78(1)
		U_3O_8	4.1387(3)	11.8449(8)	6.8315(6)	334.90(4)	22(1)
**20**	823	U_3O_8	4.1374(3)	11.8467(8)	6.8351(6)	335.02(5)	14.9(8)
		U_3O_{8-x}	4.1401(2)	11.8736(4)	6.7918(2)	333.87(2)	85.1(8)
**21**	848	U_3O_{8-x}	4.1396(1)	11.8699(2)	6.7955(2)	333.91(1)	100
**22**	873	U_3O_{8-x}	4.1391(1)	11.8684(2)	6.7968(2)	333.89(1)	100
**23**	898	U_3O_{8-x}	4.1385(1)	11.8664(2)	6.7983(2)	333.86(1)	100
**24**	923	U_3O_{8-x}	4.1383(1)	11.8648(2)	6.7989(2)	333.82(1)	100
**25**	948	U_3O_{8-x}	4.1409(2)	11.8773(5)	6.7949(3)	334.19(3)	100
**26**	973	U_3O_{8-x}	4.14558(8)	11.9524(2)	6.7638(1)	335.14(1)	100
**27**	998	UO_2	5.4714(3)			163.80(3)	3.06(9)
		U_3O_{8-x}	4.14559(8)	11.9629(2)	6.7591(1)	335.20(1)	96.94(9)
**28**	1023	UO_2	5.4817(1)			164.72(1)	10.0(2)
		U_3O_{8-x}	4.14510(8)	11.9605(2)	6.7621(1)	335.25(1)	90.0(2)
**29**	1048	UO_2	5.48360(8)			164.891(7)	24.9(6)
		U_3O_{8-x}	4.14458(9)	11.9570(2)	6.7656(1)	335.28(1)	75.1(6)
**30**	1073	U_3O_{8-x}	4.1444(1)	11.9566(2)	6.7681(2)	335.38(1)	59.8(6)
		UO_2	5.48536(7)			165.050(6)	40.2(6)
**31**	1098	UO_2	5.48741(6)			165.235(6)	58.4(6)
		U_3O_{8-x}	4.1441(1)	11.9563(3)	6.7705(2)	335.46(2)	41.6(6)
**32**	1123	UO_2	5.48956(6)			165.429(5)	78.1(5)
		U_3O_{8-x}	4.1439(2)	11.9566(4)	6.7724(3)	335.55(2)	21.9(5)
**33**	1148	U_3O_{8-x}	4.1444(3)	11.954(1)	6.7743(9)	335.62(6)	5.7(3)
		UO_2	5.49182(6)			165.634(5)	94.3(3)
**34**	1173	UO_2	5.49487(6)			165.910(5)	100
**35**	1148	UO_2	5.49786(8)			166.181(7)	100
**36**	1123	UO_2	5.5017(2)			166.53(1)	96.1(5)

		UO_{2+x}	5.4920(3)		165.65(3)	3.9(5)
**37**	1098	UO_2	5.5019(1)		166.54(1)	94.4(6)
		UO_{2+x}	5.4916(4)		165.61(4)	5.6(6)
**38**	1073	UO_{2+x}	5.492(1)		165.65(9)	7(1)
		UO_2	5.5010(2)		166.47(1)	93(1)
**39**	1048	UO_2	5.4992(1)		166.31(1)	93.4(8)
		UO_{2+x}	5.4893(8)		165.41(7)	6.6(8)
**40**	1023	UO_2	5.4976(1)		166.16(1)	93.6(6)
		UO_{2+x}	5.4870(6)		165.19(5)	6.4(6)
**41**	998	UO_2	5.4962(1)		166.03(1)	92(1)
		UO_{2+x}	5.4855(6)		165.06(5)	8(1)
**42**	973	UO_2	5.4947(1)		165.89(1)	88(1)
		UO_{2+x}	5.4835(5)		164.88(5)	12(1)
**43**	948	UO_2	5.4933(2)		165.77(2)	82(2)
		UO_{2+x}	5.4827(6)		164.81(5)	18(2)
**44**	923	UO_{2+x}	5.4805(4)		164.61(3)	19(2)
		UO_2	5.4917(2)		165.62(2)	81(2)
**45**	898	UO_2	5.4899(2)		165.46(2)	79(2)
		UO_{2+x}	5.4786(3)		164.44(3)	21(2)
**46**	873	UO_2	5.4882(3)		165.31(3)	77(2)
		UO_{2+x}	5.4773(3)		164.33(3)	23(2)
**47**	848	UO_2	5.4843(5)		164.96(4)	77(2)
		UO_{2+x}	5.4744(2)		164.06(2)	23(2)
**48**	823	UO_{2+x}	5.4720(1)		163.85(1)	25(2)
		UO_2	5.4793(4)		164.50(4)	75(2)
**49**	798	UO_{2+x}	5.47009(8)		163.676(7)	100
**50**	773	UO_{2+x}	5.46646(7)		163.350(6)	100
**51**	748	UO_{2+x}	5.46364(6)		163.097(6)	100
**52**	723	UO_{2+x}	5.46157(6)		162.912(6)	100
**53**	698	UO_{2+x}	5.46000(6)		162.771(6)	100
**54**	673	UO_{2+x}	5.45826(7)		162.616(6)	100
**55**	648	UO_{2+x}	5.45646(7)		162.455(7)	100
**56**	623	UO_{2+x}	5.45477(8)		162.304(7)	100
**57**	598	UO_{2+x}	5.45297(8)		162.143(7)	100
**58**	573	UO_{2+x}	5.45131(9)		161.995(8)	100
**59**	548	UO_{2+x}	5.44948(9)		161.832(8)	100
**60**	523	UO_{2+x}	5.44782(9)		161.684(8)	100
**61**	498	UO_{2+x}	5.4461(1)		161.535(9)	100
**62**	473	UO_{2+x}	5.4446(1)		161.402(9)	100
**63**	448	UO_{2+x}	5.4430(1)		161.258(9)	100
**64**	423	UO_{2+x}	5.4416(1)		161.133(9)	100
**65**	398	UO_{2+x}	5.4400(1)		160.99(1)	100
**66**	373	UO_{2+x}	5.4387(1)		160.871(9)	100
**67**	348	UO_{2+x}	5.4373(1)		160.753(9)	100
**68**	323	UO_{2+x}	5.4362(1)		160.652(9)	100

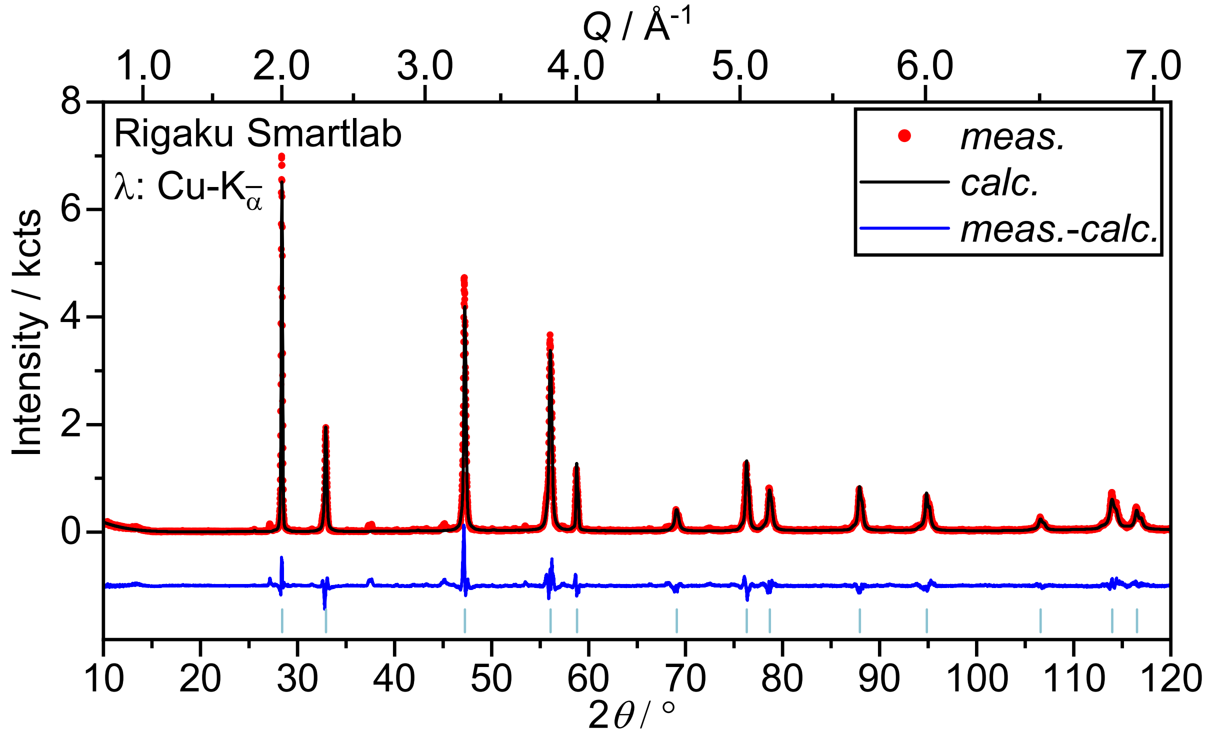

FigureS9: Rietveld refinement of the crystal structure of UO_2 based on X-ray diffraction data collected after annealing U_3O_8 in a dynamic vacuum (R_{wp} = 16.23 %, *GoF* = 2.43). Bragg markers denote from top to bottom: UO_2 ($Fm\bar{3}m$, R_{Bragg} = 6.291 %, *a* = 5.43529(8) Å, 100 wt.-%). Additional reflections and problems in the refinement of the structure model used herein point towards other or additional phases that could not be accounted for in the Rietveld analysis.

7. Reduction of U_3O_8 by CaH_2

Table S7: Refined lattice parameters and phase fractions of all phases appearing in the *in situ* X-ray diffraction experiment of the reduction of U_3O_8 by CaH_2.

ID	T / K	phase	a / Å	b / Å	c / Å	V / Å^3	ω / wt.-%
**0**	322	U_3O_8	4.1447(7)	11.925(2)	6.729(1)	332.56(1)	100
**1**	347	U_3O_8	4.1460(7)	11.913(2)	6.739(1)	332.85(9)	100
**2**	372	U_3O_8	4.1447(8)	11.894(2)	6.748(1)	332.7(1)	100
**3**	397	U_3O_8	4.1454(8)	11.878(2)	6.758(1)	332.8(1)	100
**4**	422	U_3O_8	4.1453(8)	11.857(2)	6.768(1)	332.7(1)	100
**5**	447	U_3O_8	4.1424(7)	11.838(2)	6.775(1)	332.23(1)	100
**6**	472	U_3O_8	4.1405(7)	11.829(2)	6.777(1)	331.9(1)	100
**7**	497	U_3O_8	4.1420(8)	11.850(2)	6.773(1)	332.4(1)	100
**8**	522	U_3O_8	4.1478(7)	11.909(2)	6.749(1)	333.37(9)	100
**9**	547	U_3O_8	4.1467(7)	11.936(2)	6.734(1)	333.29(9)	76(6)
		UO_2	5.468(1)			163.5(1)	24(6)
**10**	572	U_3O_8	4.147(1)	11.943(4)	6.730(2)	333.3(2)	36(1)
		UO_2	5.472(2)			163.9(1)	64(1)
**11**	597	U_3O_8	4.146(3)	11.96(1)	6.732(8)	333.7(6)	10(5)
		UO_2	5.475(3)			164.1(3)	90(5)
**12**	622	CaO	4.843(7)			113.6(5)	5(2)
		UO_2	5.484(2)			164.9(2)	95(2)
**13**	647	CaO	4.835(3)			113.0(2)	10(3)
		UO_2	5.482(2)			164.7(2)	90(3)
**14**	672	CaO	4.839(4)			113.3(2)	10(3)
		UO_2	5.484(2)			164.9(2)	90(3)
**15**	697	CaO	4.838(2)			113.2(1)	5(1)
		UO_2	5.487(2)			165.2(2)	95(1)
**16**	722	CaO	4.842(2)			113.5(2)	7(2)
		UO_2	5.489(2)			165.4(2)	93(2)
**17**	747	CaO	4.840(2)			113.4(2)	10(2)
		UO_2	5.488(2)			165.3(1)	90(2)
**18**	772	CaO	4.841(2)			113.5(1)	9(2)
		UO_2	5.489(2)			165.4(1)	91(2)
**19**	797	CaO	4.847(2)			113.8(1)	8(1)
		UO_2	5.492(2)			165.6(2)	92(1)
**20**	822	CaO	4.845(2)			113.7(1)	8(1)
		UO_2	5.493(2)			165.8(2)	92(1)
**21**	847	CaO	4.847(2)			113.9(1)	11(2)
		UO_2	5.495(2)			166.0(1)	89(2)
**22**	872	CaO	4.850(1)			114.07(1)	8(1)
		UO_2	5.498(1)			166.2(1)	92(1)
**23**	882	CaO	4.850(2)			114.1(1)	8(1)
		UO_2	5.500(2)			166.4(2)	92(1)
**24**	892	CaO	4.850(1)			114.1(1)	8(1)
		UO_2	5.500(2)			166.4(2)	92(1)
**25**	902	CaO	4.850(2)			114.1(1)	9(2)
		UO_2	5.500(1)			166.4(1)	91(2)
**26**	912	CaO	4.849(1)			114.04(1)	8(1)
		UO_2	5.499(2)			166.3(2)	92(1)
**27**	922	CaO	4.852(2)			114.2(1)	10(2)
		UO_2	5.501(2)			166.5(1)	90(2)
**28**	932	CaO	4.853(1)			114.3(1)	8(2)
		UO_2	5.501(2)			166.5(1)	92(2)
**29**	942	CaO	4.854(1)			114.3(1)	9(1)
		UO_2	5.502(2)			166.5(2)	91(1)
**30**	952	CaO	4.855(2)			114.4(1)	9(1)
		UO_2	5.501(2)			166.4(2)	91(1)
**31**	962	CaO	4.854(1)			114.37(9)	9(1)
		UO_2	5.501(1)			166.5(1)	91(1)
**32**	972	CaO	4.857(2)			114.5(1)	9(2)
		UO_2	5.502(2)			166.6(2)	91(2)
**33**	982	CaO	4.855(1)			114.45(1)	8(1)
		UO_2	5.503(2)			166.6(2)	92(1)
**34**	992	CaO	4.855(1)			114.45(8)	7.8(1)
		UO_2	5.504(2)			166.7(1)	92(1)
**35**	1002	CaO	4.857(1)			114.60(1)	9(1)
		UO_2	5.503(2)			166.7(2)	91(1)
**36**	1012	CaO	4.857(1)			114.55(9)	7(1)
		UO_2	5.504(2)			166.7(1)	93(1)
**37**	1022	CaO	4.859(1)			114.7(1)	10(2)
		UO_2	5.506(2)			166.9(1)	90(2)
**38**	1032	CaO	4.861(1)			114.85(9)	9(1)
		UO_2	5.509(1)			167.2(1)	91(1)
**39**	1042	CaO	4.861(1)			114.9(1)	8(1)
		UO_2	5.509(2)			167.2(2)	92(1)
**40**	1052	CaO	4.861(2)			114.9(1)	7(1)
		UO_2	5.508(2)			167.1(2)	93(1)
**41**	1062	CaO	4.863(1)			115.00(8)	7(1)
		UO_2	5.509(1)			167.2(1)	93(1)
**42**	1072	CaO	4.862(1)			114.96(9)	6(1)
		UO_2	5.509(2)			167.2(1)	94(1)
**43**	1073	CaO	4.862(1)			114.94(9)	8(1)
		UO_2	5.508(1)			167.1(1)	92(1)
**44**	1073	CaO	4.863(1)			114.98(1)	7(1)
		UO_2	5.509(1)			167.2(1)	93(1)
**45**	1073	CaO	4.863(1)			114.99(9)	6(1)
		UO_2	5.510(1)			167.3(1)	94(1)
**46**	1073	CaO	4.861(1)			114.85(9)	7(1)
		UO_2	5.510(1)			167.3(1)	93(1)
**47**	1073	CaO	4.861(1)			114.89(9)	7(1)
		UO_2	5.509(2)			167.2(1)	93(1)

**48**	1073	CaO	4.859(1)			114.71(9)	7(1)
		UO_2	5.507(1)			167.0(1)	93(1)
**49**	1073	CaO	4.859(1)			114.72(9)	8(1)
		UO_2	5.506(1)			166.9(1)	92(1)
**50**	1073	CaO	4.862(1)			114.90(9)	6.8(1)
		UO_2	5.510(2)			167.3(1)	93.2(1)
**51**	1073	CaO	4.861(2)			114.9(1)	7(1)
		UO_2	5.509(2)			167.2(1)	93(1)
**52**	1073	CaO	4.860(2)			114.8(1)	9(2)
		UO_2	5.508(2)			167.1(2)	91(2)
**53**	1073	CaO	4.861(1)			114.85(1)	7(1)
		UO_2	5.509(2)			167.2(2)	93(1)
**54**	299	CaO	4.810(1)			111.3(1)	7.2(1)
		UO_2	5.459(2)			162.7(2)	92.8(1)

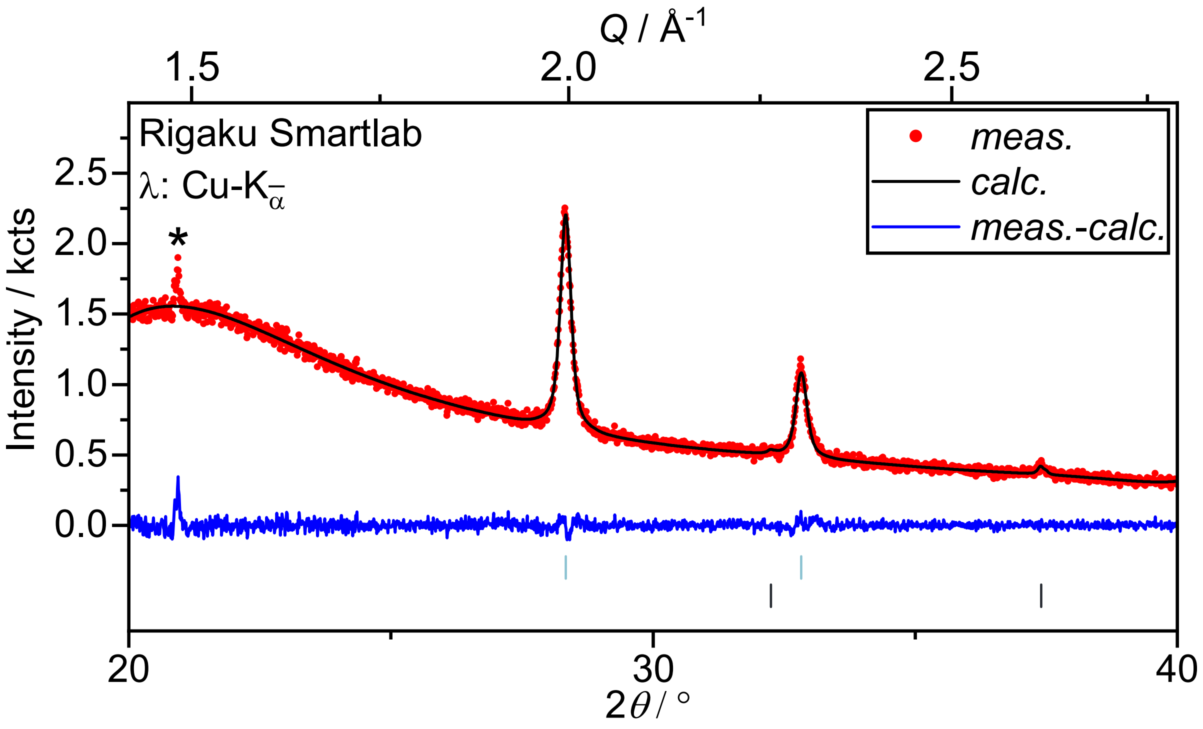

Figure S10: Rietveld refinement of the crystal structure of UO_2 and CaO based on X-ray diffraction data collected after reducing U_3O_8 by CaH_2 (R_{wp} = 3.71 %, *GoF* = 1.04). Bragg markers denote from top to bottom: UO_2 ($Fm\bar{3}m$, R_{Bragg} = 0.428 %, *a* = 5.4591(19) Å, 92.8(10) wt.-%), CaO ($Fm\bar{3}m$, R_{Bragg} = 0.837 %, *a* = 4.8102(14) Å, 7.2(10) wt.-%); asterisk (*) denotes an artifact of the measurement and does not correspond to any phase observed in the experiment.
